# Immunometabolism in rheumatoid arthritis: mechanisms, biomarkers, and the path to precision medicine

**DOI:** 10.3389/fimmu.2026.1691946

**Published:** 2026-03-06

**Authors:** Xinyu Liu, Jiajia Wang, Tong Lou, Qiaomu Tang, Zixin Fang, Wenfeng Zhang, Yinghua Hu

**Affiliations:** 1 School of Basic Medical Sciences, Changchun University of Chinese Medicine, Changchun, China; 2 College of Acupuncture and Tuina, Changchun University of Chinese Medicine, Changchun, China

**Keywords:** clinical applications, immunometabolism, personalized medicine, rheumatoid arthritis, rheumatoid arthritis (RA)

## Abstract

Synovitis and gradual joint degeneration are hallmarks of rheumatoid arthritis (RA), a chronic inflammatory illness. To sustain inflammation and tissue damage, pathogenic immune and stromal cells undergo specific metabolic reprogramming that alters glycolysis, oxidative phosphorylation, and lipid metabolism. This pathology is driven by immunometabolic dysregulation, according to new research. This review summarizes the current understanding of cell-type-specific immunometabolic dysregulation in RA, with particular attention to T cells, macrophages, B cells, and fibroblast-like synoviocytes. We assess how high-dimensional biomarkers, such as blood-based metabolomic, transcriptomic, and proteomic signatures, and synovial molecular pathotypes can be used to stratify patients and predict how well biologic and targeted treatments will work. We also reviewed treatment approaches targeting immunometabolic pathways, including new metabolic inhibitors and drug repurposing, such as metformin. To facilitate a more individualized and efficient RA treatment, we conclude by offering a clinically applicable paradigm to integrate immunometabolic profiling into precision medicine.

## 1 Introduction

Immunometabolism (IM), the interdisciplinary field examining the interplay between cellular metabolism and immune function, has revolutionized our understanding of immune-mediated diseases (1). Far from being passive responders, immune cells (ICs) actively remodel their metabolic pathways, shifting between glycolysis, oxidative phosphorylation (OXPHOS), and fatty acid oxidation (FAO), to fuel their activation, proliferation, and effector functions (2–5). In autoimmune pathology, this typically transient metabolic reprogramming becomes dysregulated, evolving into a sustained, pro-inflammatory state that actively perpetuates disease (3, 6–8).

Because of the significant, well-documented metabolic rewiring that occurs inside its target tissue, the synovium, rheumatoid arthritis (RA) is a classic paradigm for researching pathogenic IM (9–11). RA is a chronic, systemic autoimmune disease marked by synovial inflammation, autoantibody production, and progressive joint deterioration (12–15). Its pathogenesis involves a complex interplay of genetic susceptibility, environmental triggers, and dysregulated immune responses (16). A self-sustaining inflammatory cycle is established in the synovium when resident and infiltrating ICs collaborate with activated stromal cells, such as fibroblast-like synoviocytes (FLS), to create a distinct microenvironment characterized by nutrient competition, hypoxia, and the accumulation of metabolic intermediates (17, 18). This microenvironment, in turn, causes additional metabolic adjustments in a cell-type-specific way. For instance, RA T cells acquire mitochondrial malfunction that encourages a hyper-inflammatory “TNF super-producer” phenotype, while synovial macrophages adopt a permanent pro-inflammatory state supported by glycolytic reprogramming and signaling via hypoxia-inducible factor-1α (HIF-1α) and STAT3 (19–22). Bone erosion, tissue invasion, and synovitis are all directly facilitated by these adaptations.

Importantly, rather than being merely a bystander effect, immune and stromal cell metabolic rewiring is now identified as a major pathogenic driver in RA (23, 24). These modifications produce distinct cellular “metabolic signatures” that actively promote inflammation and joint destruction. These signatures are typified by changes in glycolysis, mitochondrial activity, and amino acid metabolism (24–26). This significant metabolic heterogeneity is the basis for the considerable clinical variability in RA and poses a challenge for therapy selection. Despite an increasing array of biologic and targeted synthetic disease-modifying antirheumatic drugs (DMARDs), many patients do not respond sufficiently to initial treatment, leading to a “trial-and-error” approach that hinders effective disease management (27). This highlights the pressing need for precision medicine (PM) strategies that leverage biomarkers indicative of underlying molecular and metabolic pathophysiology to predict treatment response and inform optimal therapeutic choices (27).

**Figure 1:**
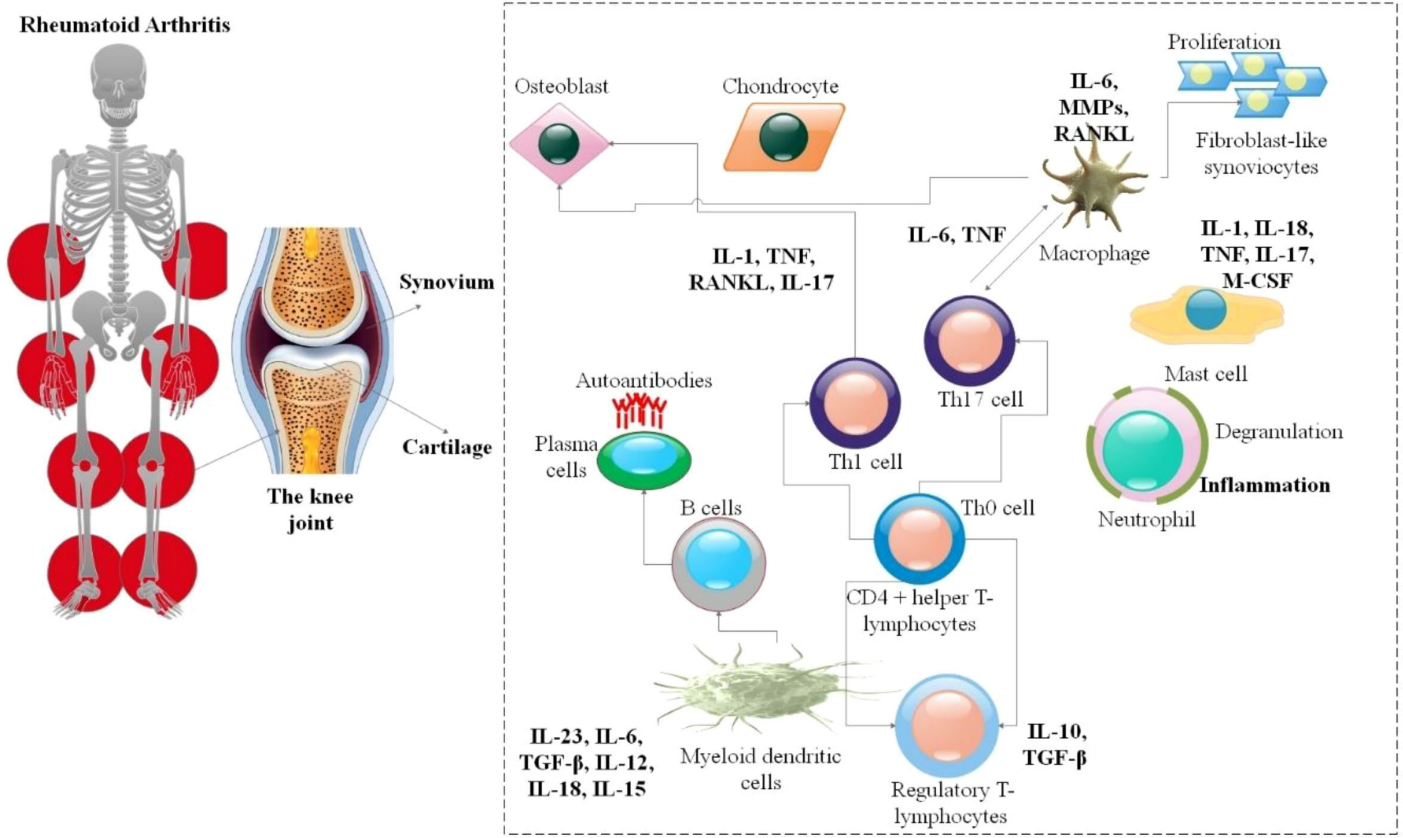
Immunometabolic interactions in the synovium of RA. The main pathogenic cell types and the paracrine signaling networks that support inflammation and joint degradation are highlighted in this schematic. Each cell type undergoes metabolic reprogramming due to the hypoxic, nutrient-poor synovial milieu (details not included in the simplified picture; see full text): pro-inflammatory cytokines, including TNF, IL-1β, IL-6, and receptor activator of nuclear factor kappa-B ligand (RANKL), are produced by activated macrophages, which promote osteoclastogenesis and a glycolytic (M1) state. Differentiating under the influence of cytokines released from dendritic cells (DCs) (IL-23, IL-6, TGF-β), T helper cells (Th1, Th17) release IFN-γ, and IL-17, which further stimulate stromal cells and promote aerobic glycolysis. Fueled by altered glucose and choline metabolism, FLS become hyperplastic and invasive, generating pro-inflammatory mediators (IL-6, IL-8) and matrix-degrading enzymes. Under the influence of RANKL and macrophage colony-stimulating factor, osteoclasts differentiated from monocytes and resorb bone, a process that requires active mitochondrial OXPHOS. Each cell type has dysregulated metabolic pathways, such as glutaminolysis in FLS, OXPHOS in osteoclasts, and glycolysis in macrophages and T cells, which both contribute to and result from this inflammatory network, creating self-replicating cycles of harm (31).

The R4RA biopsy-driven trial showed that molecular stratification of synovial tissue (ST) could inform treatment selection, and while tocilizumab (anti-IL-6R) and rituximab (anti-CD20) showed similar efficacy in patients with a lympho-myeloid pathotype, those with a molecularly defined B-cell-poor synovium responded significantly better to tocilizumab (50%) than to rituximab (12%). These findings demonstrate the validity of this PM approach (28, 29). Additionally, a poor response to both medications was linked to the pauci-immune/fibroid pathotype, indicating a treatment-resistant endotype (28). These results demonstrate that actionable biomarkers can be obtained through molecular dissection of the target tissue. Complementary initiatives aim to identify correlative, less-invasive blood-based multi-omic signatures to predict response to medicines such as anti-TNF and anti-IL-6R drugs (30) (**Figure 1**).

This review summarizes the state of the art in IM for RA from a PM perspective. First, we will describe how RA pathogenesis is maintained by cell-type-specific metabolic reprogramming. The potential of biomarkers originating from this dysregulation, ranging from peripheral blood signatures to invasive synovial pathotypes, for patient stratification and treatment prediction will then be assessed. Next, we reviewed therapeutic approaches targeting immunometabolic pathways, including novel inhibitors and drug repurposing, such as metformin, and considered how biomarkers might guide their application. Finally, we will present a conceptual framework to incorporate immunometabolic assessment into therapeutic decision-making, emphasizing key translational hurdles and future approaches to achieving more personalized care for RA.

## 2 Cell-type-specific immunometabolic dysregulation in rheumatoid arthritis

The inflamed synovium in RA is not only a site of immune cell infiltration; it is also a distinct, metabolically competitive niche that actively forms and is influenced by the pathologic processes within it. Unlike the temporary metabolic alterations observed in acute immune responses, the synovial milieu in RA, characterized by prolonged hypoxia, pathological angiogenesis, and severe competition for resources, drives a maladaptive, self-perpetuating immunometabolic reprogramming (32–34). This leads to cell-type-specific metabolic “lesions” that are important to RA pathogenesis, separating it from a generic inflammatory response and affording disease-specific treatment options.

Although basic immunometabolic principles—such as the dependence of regulatory subsets on OXPHOS and pro-inflammatory T cells on glycolysis—apply, the RA synovium imposes certain limitations and establishes particular interdependencies. Chronic hypoxia, for example, stabilizes HIF-1α, which directly skews the Th17/Treg balance, increases fibroblast invasiveness, and promotes glycolysis (19, 20, 35). Additionally, metabolic reprogramming in RA frequently results in the buildup of particular signaling metabolites, such as citrate and succinate, which function as powerful inducers of tissue damage and inflammation in this setting, generating feed-forward loops that are not always present in other autoimmune settings (21, 22, 32–34).

**Figure 2:**
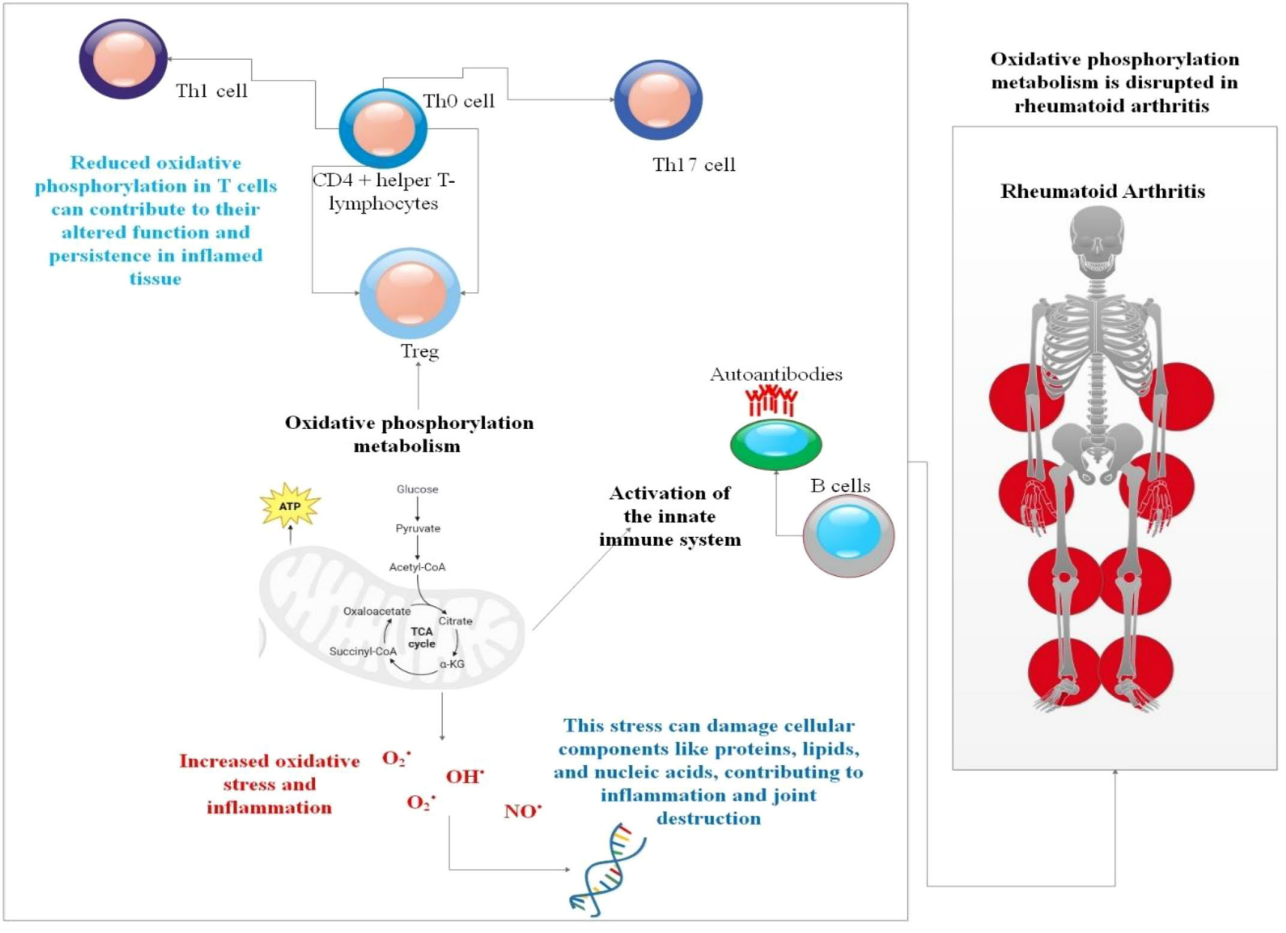
In RA, inflammation and T-cell persistence are driven by mitochondrial dysfunction. This diagram shows how pathogenic reprogramming results from reduced OXPHOS in RA CD4+ T cells. Mitochondrial failure generates a bioenergetic crisis, metabolic rewiring, and increased oxidative stress. A TNF super-producer phenotype, increased tissue invasiveness, and altered differentiation favoring pro-inflammatory Th1 cells over Tregs are all direct outcomes of these defects. Furthermore, autoreactive T-cell clones are more likely to survive and remain in the synovium due to this metabolic dysfunction. Ultimately, these defective T cells systemically exacerbate inflammation by activating B cells and innate ICs, establishing a feed-forward loop that promotes autoantibody production and joint destruction (39, 40).

With changes in glycolysis, the tricarboxylic acid (TCA) cycle, and amino acid metabolism correlated with disease activity, metabolomic profiling validates that RA presents both systemic and local metabolic disruptions (36–38). However, the essential pathophysiological understanding comes from analyzing how diverse synovial cell types, macrophages, T cells, B cells, FLS, and DCs, individually alter their metabolism to survive and prosper in this harsh environment. These adaptations are not discrete occurrences but comprise a network of metabolic symbiosis and competition (**Figure 2**)—for example, glycolytic macrophages create lactate that may fuel oxidative metabolism in FLS, whereas FLS-derived substances maintain macrophage survival (41). The pathogenic function is ultimately determined by this cell-type-specific reprogramming, which encourages tissue invasion, bone resorption, autoantibody production, and cytokine hyperproduction.

Therefore, the following sections detail the distinctive immunometabolic features of key cellular players in RA. Researchers emphasized how the distinct metabolic adaptations found in the RA synovium—such as choline kinase-driven invasion in FLS or mitochondrial dysfunction in T cells that results in a “TNF super-producer” phenotype—represent key disease mechanisms and susceptible sites for precision therapeutic intervention (3, 7, 42, Perl, 43).

### 2.1 T cells and their immunometabolism in rheumatoid arthritis

T lymphocytes are among the leading causes of RA pathogenesis. Transcriptionally distinct T-cell subsets interact intricately within the synovium: pathogenic peripheral helper and Th17 cells release cytokines (such as IL-17 and IL-21) that stimulate macrophages and synovial fibroblasts and encourage B cells to produce autoantibodies—osteoclast-mediated bone degradation results from this activation, which stimulates RANKL expression. Through immunosuppressive processes, Tregs oppose this process, but in the RA joint, their activity is usually overpowered (44, 45).

Furthermore, the pathogenic significance of CD8+ T lymphocytes is shown by the genetic connection between RA and major histocompatibility complex class I alleles. Clonally expanded CD8+ T lymphocytes that target citrullinated antigens develop in the synovium and produce cytotoxic molecules, including granzymes (Granzyme B, GZMK), in RA patients, particularly those who are anti-citrullinated protein antibodies (ACPAs)-positive. These cells have tissue-homing and pro-inflammatory characteristics, and even at-risk people have CXCR5+ fractions. Functional investigations indicate that these CD8+ T cells are directly linked to synovitis and joint deterioration, as citrullinated antigens presented by human leukocyte antigen class I induce clonal growth and the release of cytotoxic mediators (46).

Tregs restrict immune responses in three main ways: by secreting cytokines (IL-10, TGF-β, and IL-35), by directly inhibiting cell contact with molecules like cytotoxic T-lymphocyte-associated protein-4, LAG-3, and PD-1, and by disrupting target cell metabolism with enzymes like CD39/CD73. The reduced expression of reduced G-protein-signaling modulator 2 and impaired IL-6 signaling are two factors that affect Treg migration and function in RA and contribute to disease persistence. Immune homeostasis in RA may be restored by therapeutic approaches such as glucocorticoids, traditional DMARDs, and low-dose (LD) IL-2, which increase Treg numbers or improve their suppressive capacity (45). HIF-1α, which promotes inflammation, angiogenesis, and tissue destruction, is activated by hypoxia in the RA synovium. Crucially, HIF-1α also creates a pro-inflammatory feed-forward loop by stimulating Th17 cell production and reducing Treg function by inducing RORγt and degrading Forkhead box P3 (47). This Th17/Treg axis metabolic imbalance may start early in the course of the illness. Treg cells are essential metabolic sensors that respond well to local metabolic cues. This is shown by their functional adaptability and abundance in the metabolically active lamina propria of the gut. There the gut microbiota transforms dietary components into compounds that modulate immunological tolerance and Treg function. According to this, gut dysbiosis during the pre-RA phase may alter local metabolite profiles, impair Treg-mediated tolerance, and cause immunological homeostasis to collapse before the onset of clinical arthritis (47).

RA T cells exhibit a profound and specific metabolic lesion centered on mitochondrial dysfunction (21). Unlike healthy activated T cells that upregulate glycolysis to support effector functions, RA T cells have impaired mitochondrial OXPHOS. This results in low ATP generation, reduced TCA cycle activity, and the accumulation of metabolites such as citrate. The impaired malate-aspartate shuttle and endoplasmic reticulum stress lead to the pathological accumulation of TNF-α messenger RNA, converting these cells into “TNF super-producers” (21). This mitochondrial failure is linked to defective mitochondrial DNA repair. Furthermore, citrate accumulation promotes cytoskeletal hyperacetylation, enhancing T cell migration and tissue invasiveness. These metabolic alterations, governed upstream by the phosphoinositide 3-kinase (PI3K)/AKT/mTOR and HIF-1α signaling axes, lock RA T cells into a hyper-inflammatory, tissue-destructive state while making them prone to pro-inflammatory forms of cell death that release mitochondrial damage-associated molecular patterns (21, 48) (**Figure 2**).

### 2.2 Monocytes/macrophages and their immunometabolism in rheumatoid arthritis

In RA, inflammation and joint destruction are caused mainly by monocytes and macrophages. Inflammatory cytokines, chemokines, and matrix metalloproteinase (MMP) are produced by CD68+ macrophages in the synovium, and their quantity is highly correlated with radiographic progression and disease activity. Importantly, a decrease in synovial sublining macrophages is a reliable indicator of successful treatment (49).

With an increase in the intermediate CD14++CD16+ fraction, peripheral blood monocytes in RA have an activated phenotype. A worse response to methotrexate (MTX) and more aggressive illness are associated with this subpopulation. This similar intermediate population is abundant in the synovial fluid, perhaps due to local inflammatory stimuli such as TGF-β. Anti-TNF treatment may also affect a newly identified CD14^bright^CD56+ subpopulation that is elevated in RA blood and exhibits elevated inflammatory responses. Overall, the distribution and phenotype of the monocyte/macrophage subset are compartment-specific and reflect treatment outcomes and RA disease activity (49, 50).

Through significant metabolic change, macrophages, key effector cells in the pathophysiology of RA, drive inflammation and tissue death. To satisfy the bioenergetic and metabolic requirements of their pro-inflammatory (M1) state, macrophages switch from OXPHOS to aerobic glycolysis in the hypoxic, nutrient-poor synovial environment. HIF-1α controls this glycolytic switch by upregulating glucose transporters and glycolytic enzymes. Additionally, mitochondrial dysfunction, such as impaired mitophagy and accumulation of mitochondrial reactive oxygen species (mtROS), reinforces this switch by increasing NF-κB-mediated production of TNF-α and IL-1β (35, 51, 52).

Importantly, this inflammatory cascade is coordinated by certain immunometabolites. While citrate buildup promotes phospholipid and NO production, disruption of the TCA cycle results in the accumulation of succinate and itaconate, which stabilize HIF-1α and stimulate IL-1β expression. Anti-inflammatory (M2) macrophages, on the other hand, rely on FAO and OXPHOS, which are mechanisms enhanced by IL-4/IL-13 signaling via peroxisome proliferator-activated receptor gamma coactivator 1-beta. Moreover, increased choline phosphorylation and absorption in RA macrophages promote membrane biosynthesis and cytokine production. At the same time, arginine metabolism, which is divided between the arginase and nitric oxide synthase pathways, controls mitochondrial fission and mtROS generation. The integration of these metabolic adaptations—FAO/OXPHOS maintaining M2 function and glycolysis promoting M1 polarization—via STAT3-dependent signaling makes macrophage IM a viable therapeutic target for repolarization in RA (35).

Monocytes and macrophages in the RA synovium are skewed towards a pro-inflammatory, glycolytic (M1-like) phenotype, a shift driven by hypoxia, cytokines (e.g., IFN-γ, TNF-α), and metabolic sensors (41). Key to this reprogramming is the stabilization of HIF-1α and the activation of the STAT3 pathway, which upregulate glycolytic enzymes such as 6-phosphofructo-2-kinase/fructose-2,6-biphosphatase 3 (PFKFB3) (53, 54). This glycolytic switch supports rapid ATP production and provides intermediates for biosynthetic pathways, fueling cytokine production (IL-1β, IL-6, TNF-α) and antigen presentation. Significantly, metabolic rewiring also alters the production of immunometabolites. Succinate accumulation stabilizes HIF-1α, creating a feed-forward loop that promotes inflammation, whereas itaconate (derived from the TCA cycle) exerts anti-inflammatory effects by activating the nuclear factor erythroid 2-related factor 2 (Nrf2) pathway and inhibiting glycolysis. An itaconate deficit has been associated with worse RA disease activity, highlighting the regulatory role of these metabolites (55).

### 2.3 B cells and T follicular helper cell and their immunometabolism in rheumatoid arthritis

By promoting B-cell development and the generation of autoantibodies, Tfh cells play a crucial role in the pathogenesis of RA. HIF1α-mediated glycolysis supports the function of circulating Tfh (cTfh) cells, which are increased in RA patients and correspond with disease activity. Peripheral helper T (Tph) cells, a similar pathogenic fraction, also proliferate in RA and support B cells in inflammatory tissues, but they produce only cytotoxic mediators. Although both categories are elevated in RA, this study found that they differ metabolically and functionally: While Tph cells have a cytotoxic profile associated with increased mtROS generation, cTfh cells exhibit a high B-cell-help potential associated with increased glycolysis. These results provide a fresh understanding of the pathophysiology and potential treatment targets by revealing subset-specific metabolic processes that control distinct effector activities in RA (56).

Anti-cyclic citrullinated peptide (CCP) antibodies and rheumatoid factor (RF) autoantibodies are important serological markers for RA, a chronic synovial and systemic inflammatory disease that results in joint damage. Tfh cells aid B cells in producing these autoantibodies, highlighting their crucial pathogenic function. This is supported by the fact that RA ST has increased levels of IL-21, a cytokine released mainly by Tfh cells, and its receptor, IL-21R, and that a deficit in IL-21R prevents arthritis in mouse models (57). Researchers conducted *in vitro* functional tests and examined blood from patients and healthy controls to determine the pathogenic role of IL-21 in RA. Anti-CCP antibodies and the disease activity score (DAS28) were associated with elevated serum IL-21 levels and increased frequencies of cTfh-like cells in RA. Patients’ Tfh-like and B cells have increased levels of the IL-21 receptor (IL-21R). Functional investigations showed that B cell activation, proliferation, differentiation into plasmablasts, and IgG/IgM production were significantly increased by IL-21 stimulation; these effects were reversible upon IL-21R blockade. These findings demonstrate the IL-21/IL-21R pathway as a potential therapeutic target and identify IL-21 as a key mediator of the pathogenic Tfh-B cell axis in RA (57).

While the IM of RA B cells is less well characterized than that of T cells, their activation, proliferation, and differentiation into antibody-producing plasmablasts are highly energy-intensive processes that depend on glycolysis, glutaminolysis, and fatty acid synthesis (FAS) (58). The metabolic needs of autoreactive B cells are likely supported by Tfh cells within synovial ectopic lymphoid structures. Tfh cells, which are critical for providing help to B cells, themselves rely on increased glycolysis and mitochondrial respiration. This metabolic crosstalk is central to the generation of pathogenic autoantibodies, such as RF and ACPAs. Understanding the metabolic dependencies of the B-cell axis provides a rationale for the efficacy of B-cell-depleting therapies like rituximab. It suggests that metabolic inhibitors could synergize with such targeted approaches (37).

A thorough understanding of B-cell biology is essential for developing customized treatments for RA. Stratified management is justified since the pathogenic triad of autoantibody generation (RF/ACPA), pro-inflammatory cytokine release, and antigen presentation by B cells varies across individuals. Novel pathogenic B cell subsets are emerging from advances in single-cell multi-omics; these subsets exhibit distinct transcriptional patterns and signaling requirements (e.g., via Bruton’s tyrosine kinase (BTK), spleen-associated tyrosine kinase, or B-cell activating factor pathways). This variability in clinical response to current B-cell-depleting treatments, such as rituximab, is supported by this finding. Therefore, precision immunomodulation—the process of choosing patients based on specific autoantibody profiles or synovial pathotypes for customized interventions—is the key to the future of B-cell-targeted treatment. These might include pathway-specific inhibitors (such as BTKi) for patients with specific signaling hyperactivation, next-generation CD19/CD20 CAR-T cells for refractory disease, or methods to increase the number of regulatory B cells in patients with a weak immunoregulatory network. Additionally, tailored administration to specific B cell compartments is enabled by nanotherapeutic platforms, thereby improving safety and effectiveness. The next frontier in achieving long-lasting remission and improving long-term outcomes in RA is this customized, mechanism-based approach, which shifts from widespread depletion to precise functional modulation (59, 60).

### 2.4 Fibroblast-like synoviocytes and their immunometabolism in rheumatoid arthritis

A crucial part of the invasive synovium, FLSs play a significant part in the development and maintenance of damaging joint inflammation. By regulating the composition of synovial fluid and the extracellular matrix (ECM) of the joint lining, FLSs typically maintain the structural and dynamic integrity of joints. However, FLSs have pathogenic characteristics in RA. RA-FLSs multiply and take center stage in the damaging pannus that defines RA patients’ synovitis. Additionally, RA-FLSs have an aggressive character and mediate joint degeneration and inflammation. Due to their rapid multiplication over the course of the illness, RA-FLSs have a high ATP requirement. Similar to Th17 cells, RA-FLSs express more glucose transporter 1 (GLUT1), hexokinase 2 (HK2), pyruvate kinase M2, PFKFB3, and MCT4. Additionally, metabolomics research showed that RA-FLSs significantly boost glycolysis for ATP synthesis. RA-FLSs need glycolysis and glutaminolysis for their proliferation, survival, activation, and secretion of inflammatory cytokines, including chemokines (such as IL6 and MMP-3). At the same time, effector T cells require enhanced glycolysis for growth and effector functions (61–63).

RA FLS transform into aggressively invasive, apoptosis-resistant cells that drive pannus formation and cartilage destruction. Metabolic rewiring, multifaceted, supports this aggressive phenotype. RA FLS exhibit a “pseudo-hypoxic” state with increased glycolysis even in normoxia, partly driven by HIF-1α and pro-inflammatory cytokines (19). Notably, choline and glycerophospholipid metabolism are intensely activated. Choline kinase (Chokα) converts choline to phosphatidylcholine, activating the mitogen-activated protein kinase (MAPK)/PI3K/Akt pathway to promote proliferation, MMP expression, and invasion (26). Alterations in glycerophospholipid pathways correlate with inflammatory markers. Furthermore, FLS engage in metabolic symbiosis with macrophages; FLS can take up lactate produced by glycolytic macrophages to fuel their own metabolism, while FLS-derived factors promote macrophage survival and activation, creating a vicious, self-sustaining inflammatory cycle (41).

### 2.5 Dendritic cells and their immunometabolism in rheumatoid arthritis

Different subsets of DCs, known as plasmacytoid DCs (pDCs) and conventional DCs (cDCs), have distinct functions in the pathophysiology of RA. cDCs have enhanced antigen presentation and co-stimulatory capacity, which promote CD4+ T cell activation and the generation of pro-inflammatory cytokines. This is especially true of the CD1c+ cDC2 subgroup, which is abundant in RA synovial fluid. On the other hand, because their depletion makes arthritis worse in mice, pDCs could have preventive benefits. Although their exact regulatory role remains unknown, pDCs in synovial fluid from RA patients exhibit an immature phenotype with a low expression of co-stimulatory markers, and single-cell RNA sequencing (scRNA-seq) indicates that an inactive disease state is associated with a pDC population with a “healthy” transcriptomic profile (53, 54). Due to hypoxia and pro-inflammatory signals, cDCs in the synovium of RA show increased glycolytic activity, which supports their enhanced ability to present antigens and activate T cells. The loss of immunological tolerance is facilitated by this metabolic reprogramming, which encourages a pro-inflammatory phenotype. On the other hand, monocyte-derived DCs (moDCs) may enter a tolerogenic state by encouraging OXPHOS. Thus, a possible treatment approach to restore immunological balance and reduce synovial inflammation in RA is to target the IM of DCs, for example, by modifying their glycolytic flux (53).

**Figure 3:**
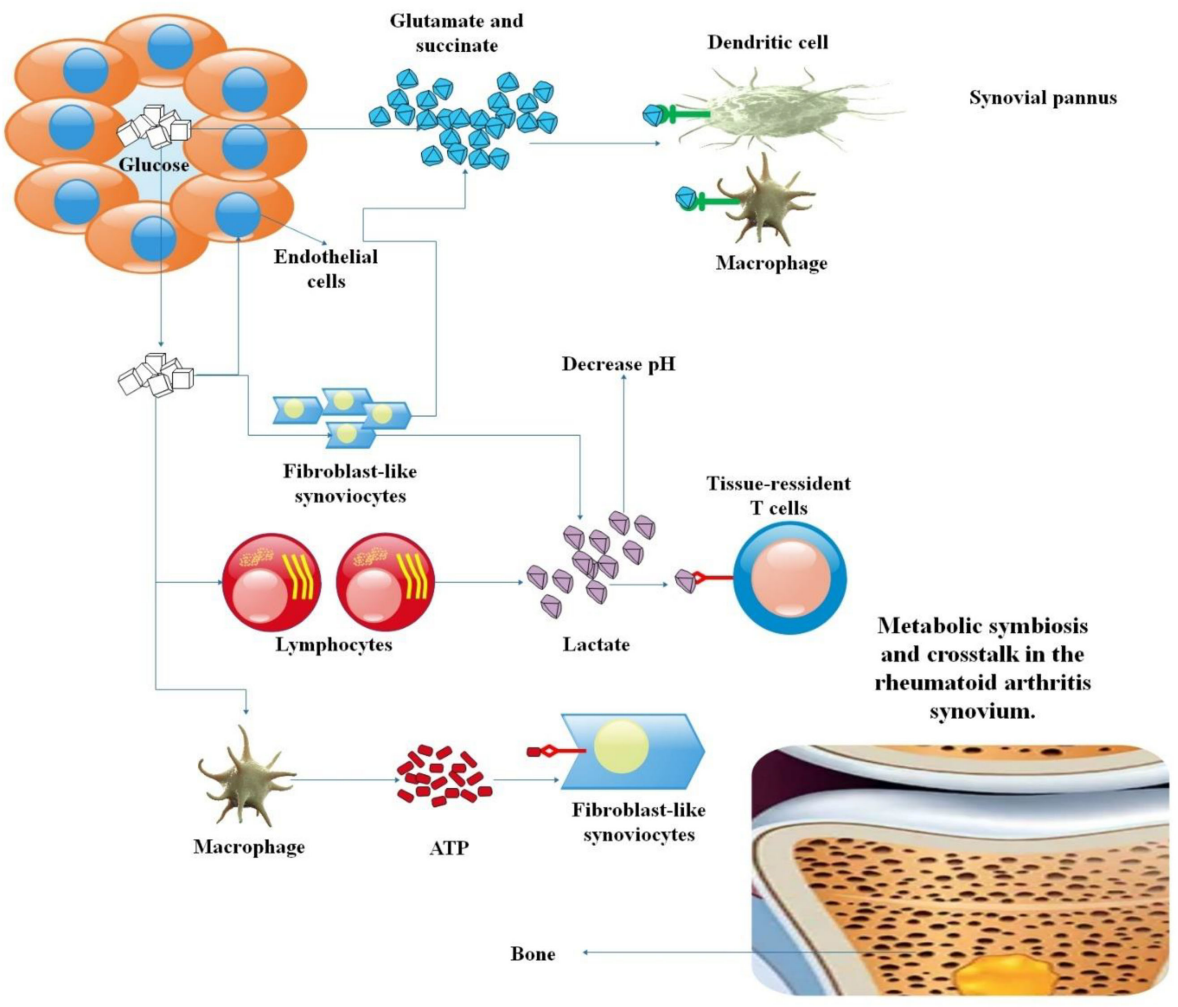
The RA synovium is fueled by metabolic symbiosis and interaction. Chronic inflammation and tissue degradation are sustained by a network of nutrient exchange driven by the hypoxic, inflammatory joint microenvironment. Glycolysis is accelerated by activated macrophages, DCs, and lymphocytes, resulting in elevated lactate levels that acidify the synovial space and exacerbate tissue damage. FLSs are propelled by this lactate and perhaps by direct ATP transfer, which facilitates the invasion, growth, and development of damaging synovial pannus. The production of pathogenic tissue-resident T cells is also promoted by this metabolic environment, and endothelial cells mediate the link between metabolic alterations and aberrant angiogenesis. Targeting these exchanges is a unique way to break the synovitis cycle, as this figure shows that immunometabolic reprogramming in RA is a collaborative process (65, 66).

DC activity is primarily determined by metabolism, with catabolism favoring tolerogenicity and anabolism fostering immunogenicity. In the hypoxic RA synovial environment, synovial cDCs and moDCs are pro-inflammatory due to decreased OXPHOS and increased glycolysis. Synovial cDC1 and cDC2 exhibit higher T cell activation, improved migratory and costimulatory ability, and elevated hypoxic and glycolytic genes. In a similar vein, moDCs treated with RA synovial supernatants produce more pro-inflammatory cytokines and exhibit increased glycolytic flux. Metabolites further shape DC activity; increased synovial succinate activates DCs via G Protein-Coupled Receptor 91, stimulating Th17 responses. On the other hand, in RA joints, pDCs, which depend more on OXPHOS, seem less inflammatory. This metabolic polarization demonstrates how the RA synovial milieu metabolically trains DCs to maintain inflammation, with glycolysis driving pathogenic cDC/moDC activity and OXPHOS potentially maintaining pDC control (17, 53, 64) (**Figure 3**).

The study of IM in RA has yielded important new insights, but it also has significant drawbacks and knowledge gaps. The majority of the evidence comes from *in vitro* research or animal models, which may not adequately represent the intricate, dynamic metabolic ecosystem of the human synovium, even as cell-specific metabolic profiles, such as those of glycolytic macrophages and mitochondrially deficient T cells, are becoming more clearly defined. A key drawback is the prevalence of correlative evidence; for instance, certain metabolite modifications are associated with disease activity, but demonstrating a direct causal role in pathogenesis or treatment resistance remains problematic. There is also a relative scarcity of data on the IM of critical actors, such as B cells and DCs, compared to T cells and macrophages. Furthermore, studies generally fail to account for the considerable variation between individuals and within ST compartments. Looking ahead, future research must emphasize spatial metabolomics to map metabolite distribution *in situ*, longitudinal multi-omics in patient cohorts to follow metabolic changes with illness progression and medication, and functional genetic screening to show causation. The essential next step is to go beyond descriptive profiling and determine whether targeting these pathways can durably reset immunological tolerance and prevent disease progression in people.

## 3 Biomarkers for patient stratification in RA: synovial pathotypes and peripheral blood signatures

Although the seropositive/seronegative dichotomy based on autoantibodies such as RF and ACPAs provides a basic clinical and prognostic stratification in RA, it is not very useful for guiding the selection of contemporary targeted therapies. Many seropositive and seronegative patients exhibit different reactions to the same biologic or targeted synthetic DMARD, underscoring the urgent need for biomarkers that reflect the underlying functional biology, such as immunometabolic state and synovial pathobiology (67, 68). Immunometabolic profiling provides a distinct stratification axis that goes beyond serological status, categorizing patients based on primary pathogenic cellular drivers and associated metabolic dependencies. For instance, a patient with a “diffuse myeloid” synovial pathotype and a related blood metabolomic profile of heightened glycolysis may have a different optimum treatment than a patient with a “lympho-myeloid” pathotype, independent of their ACPA status (28, 29, 69, 70). This section assesses biomarkers that aim to go beyond serology and provides a mechanistic, predictive map for precision therapy selection in RA, ranging from the invasive gold standard of synovial biopsy to newly developed blood-based multi-omics signatures.

### 3.1 Synovial tissue biopsy (a bedside tool for pathobiological insight and response stratification)

The primary source of disease in inflammatory arthritis is ST, characterized by immunological infiltration and hyperplasia. Recent advances in the molecular analysis of ST biopsies have revealed significant heterogeneity, offering important new insights into pathophysiology and opportunities for treatment customization. ST profiling has the potential to guide targeted drug selection, predict response, and advance PM by identifying pre- and post-treatment synovial biomarkers through high-throughput omics; however, this outcome depends on making routine synovial biopsy and analysis a standard of clinical care (71). The most straightforward way to understand the predominant local pathobiology is to examine the synovium directly. The lympho-myeloid, pauci-immune, and fibroid subtypes are molecular and histological synovial pathotypes identified by landmark investigations and represent distinct cellular infiltrates and pathway activities (71).

Researchers validated a minimally invasive, ultrasound (US)-guided synovial biopsy approach for the histological evaluation of minor hand joints in RA. In contrast to the gold standard of surgical arthrotomic biopsies, the percutaneous US-guided approach consistently produced evaluable tissue, providing enough tissue area for reliable digital image analysis. With a margin of error of less than 10%, the research found that measuring a cumulative tissue area of 2.5 mm^2^—achievable from eight randomly selected sections of US-guided samples—provided a representative assessment of key ICs (CD68+ macrophages, CD3+ T cells, and CD20+ B cells). The results demonstrate that US-guided synovial biopsy is a dependable and less invasive method to characterize synovial pathology in RA (72).

The R4RA study determined whether tocilizumab, an IL-6 inhibitor, was more effective than rituximab, a B-cell-depleting drug, in RA patients with an insufficient anti-TNF response, particularly those categorized as “B-cell-poor” based on synovial histology. Reclassification of patients using a more accurate RNA sequencing (RNA-seq)-based molecular signature revealed that those with a molecularly defined B-cell-poor synovium responded significantly better to tocilizumab than to rituximab, even though histological stratification alone did not demonstrate a significant difference in clinical response. This indicates that stratification of ST by RNA-seq has greater predictive ability than histology for guiding biologic selection. Before practical adoption, validation in other cohorts is necessary, even if the findings show promise for PM (73).

By differentiating between persistent inflammatory refractory RA (PIRRA) and non-inflammatory refractory RA (NIRRA) refractory phenotypes, compared with non-refractory RA, researchers defined the synovial pathology of refractory RA. PIRRA was associated with active lympho-myeloid and diffuse myeloid inflammation, elevated inflammatory markers, and increased power Doppler US activity. In contrast, NIRRA patients showed significantly lower synovitis scores, fewer lymphoid aggregates, and a predominance of pauci-immune/fibroid pathotypes, as determined by immunohistochemical analysis of synovial biopsies. Notably, NIRRA patients reported greater pain, more opioid usage, and worse quality of life while having similar overall disease activity levels, suggesting a substantial burden of noninflammatory diseases. These results highlight distinct synovial and clinical characteristics in refractory RA, in which fibrotic and noninflammatory processes, rather than active synovitis, are the main drivers of NIRRA (70). Inflammatory arthritis may be accurately diagnosed using the Krenn synovitis score, but treatment response cannot be predicted. ST functions as a sensitive pharmacodynamic marker, according to preliminary proof-of-mechanism experiments, and rapid changes in ICs (such as CD68+ macrophages) correlate with drug activity. These investigations made biopsy-driven stratified trials possible. The seminal R4RA experiment demonstrated that a more accurate categorization of synovial pathotypes (fibroid, diffuse myeloid, and lympho-myeloid) was possible beyond the straightforward B-cell-rich/poor dichotomy. It was shown that tocilizumab was more effective in diffuse myeloid tissues, although both rituximab and tocilizumab were beneficial in lympho-myeloid tissues. The fibroid phenotype has been associated with resistance to many drugs. While thorough transcriptome sequencing of ST identified predictive gene signatures, simple B-cell categorization failed to predict response in biologic-naïve patients, as subsequently verified by studies such as STRAP. B/Tfh-like, inflammatory myeloid, and fibroblast subsets are examples of cell clusters discovered by scRNA-seq whose molecular programs correspond to pathotypes and predict response or resistance to certain medications. In summary, combining bulk and single-cell transcriptomics with histological pathotypes offers a strong foundation to reveal the molecular heterogeneity of RA. It enables PM by matching patients to treatments according to their synovial molecular profile (74).

To learn more about the processes behind RA therapy response and resistance, researchers examined synovial samples from the R4RA PM study. It revealed several molecular endotypes: a stromal/fibroblast profile was linked to multidrug resistance, whereas humoral immune markers predicted response to rituximab and tocilizumab. Post-treatment molecular alterations demonstrated divergent drug-specific effects. Machine learning models were created using these signals to forecast multidrug resistance (area under the curve (AUC) = 0.69), tocilizumab (AUC = 0.68), and rituximab (AUC = 0.74) response. The results encourage incorporating tissue-based molecular pathology into clinical algorithms to optimize therapy selection and to develop novel therapeutics for patients with resistance, as they highlight the many molecular pathways in the synovium that influence clinical outcomes (29).

Building on these findings, individual patient responses to etanercept, tocilizumab, and rituximab can now be accurately predicted by machine learning models trained on synovial RNA sequencing data (AUC ~0.75) (75). In the direction of a single clinical decision algorithm for biologic selection, these prognostic markers have been combined into a useful 524-gene synovium-specific panel (75).

Despite this strong proof of concept, challenges remain in incorporating synovial biomarkers into standard clinical practice. Procedural invasiveness, expense, the need for specialized interdisciplinary processes, and the need for further validation in larger, more varied populations are among the challenges (29, 73). However, ST biopsy remains a vital research tool and a prospective foundation for a future PM paradigm in RA in which the molecular pathology of the target organ guides treatment.

Although the concept was successfully validated, routine clinical translation is hampered by significant limitations. First, the prediction accuracy, although superior to serology, is modest (~75%), showing that synovial molecular status is a key, but not only, predictor of response. Second, the invasive nature of biopsy, despite technological advancements, fundamentally limits scalability, patient acceptability, and repeated evaluation. Third, considerable logistical and cost challenges persist, requiring specialized inter-disciplinary teams for biopsy processing, sophisticated omics analysis, and bioinformatic interpretation, making it a tool presently restricted to research-centric tertiary institutes. Fourth, because of the dynamic nature of synovial biology, a single biopsy only provides a snapshot; the evolution of pathotypes with treatment or over time is poorly understood; the majority of validation comes from specific populations, such as anti-TNF inadequate responders; and generalizability across disease stages, ethnicities, and treatment-naïve patients requires confirmation in larger, diverse cohorts (**Box 1**).

Box 1 **Synopsis of synovial biomarker testing in RA: Clinical workflow and assay feasibility.**

1. Clinical pathway
  • Patient selection: Patients with active RA who do not respond well to standard treatment are the primary focus. They are often enrolled at a tertiary center for a PM study or for a diagnostic evaluation.
  • Timing: Taken into account before starting or transitioning to a targeted synthetic DMARD or advanced biologic (e.g., after failing a first TNF inhibitor).
  • Procedure: A needle biopsy of an actively inflammatory joint performed under the guidance of minimally invasive ultrasonography.
  • Therapeutic impact: Patient stratification based on results may help choose a course of treatment. For instance, a fibroid pathotype may indicate a multidrug-resistant phenotype, but a low synovial B-cell signature may prefer tocilizumab over rituximab.
2. Assay characteristics and feasibility
  • Tissue source: ST (invasive) vs. peripheral blood (non-invasive).
  • Analytical methods:
    • Histology/immunohistochemistry: Pathotype categorization standards (lympho-myeloid, diffuse myeloid, fibroid) set the bar high—achieving high repeatability among readers when conducted in designated facilities.
    • Molecular profiling: Gene panels for prediction (like nCounter) or bulk RNA-seq (discovery) to find predictive signatures. Excellent repeatability within established tests.
  • Workflow integration: A collaborative effort between a rheumatologist, an interventional sonographer, a pathologist, and a bioinformatician is necessary. The current hurdle to regular use is the turnaround time from biopsy to outcome.

Current status: An effective research instrument with solid clinical trial prediction data (R4RA, STRAP). Not yet considered standard of care; this is due to the need for more extensive validation, improvement of workflow, and proof of cost-effectiveness in widespread clinical practice.

### 3.2 Blood-based omics (from metabolic to molecular stratification)

To overcome the limitations of tissue biopsies, substantial effort is focused on identifying systemic blood (serum/plasma) biomarkers that reflect synovial biology and disease activity. Metabolomic profiling of blood from RA patients has advanced the identification of biomarkers for disease stratification, prognosis, and treatment prediction, yet a core challenge remains finding reliable circulating markers of real-time disease activity. Such biomarkers, spanning genomics, transcriptomics, proteomics, and metabolomics, can enhance diagnostic specificity, forecast therapeutic response, and clarify drug mechanisms, thereby guiding personalized treatment strategies. For instance, multi-biomarker panels (e.g., a 12-protein serum score) have been developed to quantify disease activity and correlate closely with clinical indices such as DAS28-C-reactive protein (CRP). Among immune players, B cells hold particular significance, underscored by the long-established roles of autoantibodies (RF, ACPA) and validated by the efficacy of B-cell-depleting therapies like rituximab. Ongoing research continues to unravel the multifaceted, antibody-independent functions of B cells, positioning them as central therapeutic targets. Integrating multi-omics biomarkers into clinical algorithms promises to reduce diagnostic ambiguity, optimize drug selection, and improve health outcomes in RA (68, 76, 77). This integrative multi-omics analysis identified distinct plasma proteomic and metabolomic patterns between ACPA-positive and ACPA-negative RA subgroups, suggesting that serostatus alone may not adequately capture disease heterogeneity. Elevated complement proteins (Complement Factor B, Complement Factor H-Related 5) and particular changes in lipid/pyrimidine metabolism were the hallmarks of ACPA-RA. These multi-omic characteristics were incorporated into a machine learning model that outperformed single-omic methods in classification accuracy (AUC > 0.90). These results demonstrate how blood-based multi-omic profiling might enhance diagnostic accuracy, improve stratification, and eventually direct therapeutic regimens tailored to individual subgroups in RA (78).

High-throughput profiling (e.g., via nuclear magnetic resonance (NMR), gas chromatography–mass spectrometry, liquid chromatography–mass spectrometry) has defined distinct metabolic fingerprints in RA patients compared to healthy controls, characterized by alterations in glycolysis, TCA cycle, amino acid, and lipid metabolism (32, 79, 80). Researchers consistently observed changes in key pathways that correlated with systemic inflammation indicators, such as CRP, including increased glycolysis, disrupted FAO, and altered amino acid and TCA cycle metabolism. Disease-specific metabolic reprogramming that promotes inflammation, IC infiltration, and tissue damage is characterized by elevated serum fatty acids and cholesterol and reduced serum glucose and amino acids. These results demonstrate that metabolomics is a powerful tool to understand the pathophysiology of RA and to identify prospective treatment targets and diagnostic markers aimed at restoring metabolic balance (32, 79, 80). Disease activity scores, such as DAS28-CRP, correlate with these markers (80, 81). More significantly, baseline metabolic profiles may predict therapeutic response—for example, metabolomic profiling using NMR and mass spectrometry shows promise in predicting therapeutic response in RA. Studies analyzing pre-treatment plasma metabolites identified distinct metabolic signatures in patients who subsequently responded or did not respond to specific biologics. For rituximab, baseline differences in lipids (phosphatidylcholines, phosphatidylserines) and polar metabolites (succinate, choline, glycine) distinguished future responders from non-responders. Similarly, in patients starting MTX, a three-metabolite panel (homocystine, glycerol-3-phosphate, 1,3-/2,3-diphosphoglyceric acid) predicted inadequate response with high accuracy (AUC 0.81). Researchers’ results suggested that treatment resistance is caused by metabolic dysregulation in pathways such as glycolysis and amino acid metabolism. By incorporating these metabolomic markers into clinical procedures, it may be possible to predict medication effectiveness early on, resulting in more individualized and successful treatment plans for RA (82, 83). These are primarily predictive biomarkers in the discovery/validation phase.

Transcriptomic analysis offers a more dynamic and tissue-specific view of disease than DNA-based methods, though RNA’s inherent instability demands meticulous sample handling. Studies comparing transcriptomes from RA synovium, blood cells, and bone marrow with those from healthy controls have revealed disease-specific expression patterns. While microarrays provided initial insights, RNA-seq now enables unbiased, genome-wide detection of transcripts—including low-abundance and novel splice variants—offering superior resolution—for example, RNA-seq of synovial fibroblasts identified hundreds of differentially expressed genes in RA, implicating pathways in cell proliferation, motility, immune activation, and lipid metabolism. As sequencing technologies advance, transcriptomics will continue to deepen our understanding of RA pathogenesis and highlight novel therapeutic targets (84). Researchers investigated the immunometabolic profile of circulating CD14+ monocytes in RA and revealed a pre-symptomatic inflammatory phenotype. Monocytes from RA patients exhibit heightened inflammatory responses, including exaggerated cytokine/chemokine production and enhanced migratory capacity. At the metabolic level, these cells demonstrate mitochondrial hyperactivation and a distinct shift toward glycolysis, driven by increased expressions of HIF1α, HK2, and PFKFB3, and dependent on glucose uptake. STAT3 regulates this pro-inflammatory glycolytic reprogramming, as STAT3 inhibition reverses both the metabolic shift and the inflammatory output. Notably, this hyperactive, metabolically dysregulated monocyte phenotype is already detectable in individuals at risk. It is reflected in their synovial gene signatures, suggesting that it may serve as an early, targetable driver of RA pathogenesis (85). To overcome the drawbacks of bulk transcriptomics in RA, researchers combined scRNA-seq with spatial proteomics via laser capture microdissection to examine the synovial lining and sublining compartments. This high-resolution method revealed unique spatial patterns of protein expression in the sick synovium, emphasizing key pathogenic proteins involved in ECM remodeling (procollagen-lysine,2-oxoglutarate 5-dioxygenase 2, osteoglycin) and membrane function (TYRO protein tyrosine kinase-binding protein, Solute Carrier Family 16 Member 3). The derivation of cellular enrichment within these niches, which revealed contributions from T cells, fibroblasts, myeloid cells, and other cell types, was made possible by integrating scRNA-seq data. In addition to revealing molecular causes with compartment-specific clarity and identifying specific protein targets for therapeutic intervention, researchers combined a spatial proteomic and transcriptomic approach to offer a fresh, comprehensive perspective on RA synovial pathophysiology (86).

By integrating computational analysis, transcriptomic data, and protein microarrays, researchers have developed and refined quantitative methods to identify and validate blood-based protein biomarkers for RA. These biomarkers are evaluated by their correlation with clinical disease activity, measured by DAS28-CRP, and their contribution to multivariate predictive models. A key achievement is the creation of a precise serum-based Multi-Biomarker Disease Activity (MBDA) score derived from 12 prioritized biomarkers that robustly correlates with and stratifies clinical disease activity into low, moderate, and high levels. This validated, algorithmic approach provides an objective molecular measure of RA disease activity, enhancing traditional clinical assessment. Serum protein concentrations are integrated into commercially available MBDA scores, which provide an objective assessment of disease activity that correlates with radiographic progression and clinical ratings (87). Although the MBDA score has achieved greater clinical integration as a validated prognostic and disease activity biomarker, further research is needed to fully understand its role as a purely predictive tool for certain medications.

To create better predictive models in the future, multi-omic data (genomics, transcriptomics, proteomics, and metabolomics) must be integrated with machine learning. Research has effectively integrated blood metabolomic and lipidomic patterns to categorize disease activity and diagnose seronegative RA with high accuracy (88). To prevent overinterpretation and clarify translational readiness, biomarkers must be evaluated on a spectrum from discovery to clinical utility (**Table 1**).

**Table 1:** Evidence grading for selected RA biomarkers.

Biomarker/Signature	Discovery evidence	Validation status	Current clinical utility/pathway	Reference
MBDA score (12-protein panel)	Stratified disease activity is strongly correlated with DAS28-CRP	Verified in a clinical setting. Food and Drug Administration-reviewed and CE-marked; used in some trials and observational studies.	Not yet common for Tx selection; this auxiliary technique measures disease activity	(87)
Rituximab response metabolites	Response is predicted by baseline lipids and polar metabolites (such as succinate and phosphatidylcholines)	Limited; needs prospective confirmation; replicated in other cohorts	Used solely for research. Care has not yet included a predictive algorithm	(82)
MTX response metabolites	Inadequate MTX response is predicted by a three-metabolite panel (AUC 0.81)	Awaiting independent confirmation. Found in the tREACH cohort; further widespread replication is required	Preclinical investigation; not applied while making clinical decisions	(83)
ACPA status proteomic/metabolomic signatures	Different plasma profiles (such as complement proteins and lipids) of ACPA+ and ACPA-RA	Finding of a single cohort; although machine learning models are pretty accurate, they need external validation	A tool for stratification in research; not a standard for diagnosis or prognosis	(78)
Seronegative RA diagnostic model	The 26-metabolite/lipid panel has an accuracy of around 90% in detecting seronegative RA	Solely internal validation; testing in a variety of multi-center cohorts is necessary	Proof of concept; not a verified diagnostic test	(88)
Fibroblast growth factor 21 (adipokine)	Links to the intensity and course of pain, regardless of body mass index	Connection based on observation; interventional studies have not shown causal or prognostic significance	Research biomarker for the pathophysiology of pain; no treatment implications as of yet	(89)
Synovial pathotypes (lympho/diffuse/fibroid)	Determined by histology and immunohistochemistry; in clinical studies, it correlates with treatment response (R4RA, STRAP)	Validated prospectively in studies using biopsy stratification; repeatable by different expert centers	Stratification tool for clinical trials. Because it is intrusive, it is not routine care	(29, 73)
Circulating Tph cells	Correlates with early RA activity and synovial inflammation	Repeated across many cohorts; correlation with the results shown	Promising biomarker for investigation; role in the study’s prediction and categorization	(90, 91)

The list of possible biomarkers for RA has grown dramatically as a result of transcriptomic and metabolomic research, which has provided stratification tools and revealed the metabolic foundations of the illness. However, most signatures are still in the discovery or validation stage, as shown in the evidence table. Although it is not currently used to select specific treatments, the MBDA score is a notable exception to the rule as a regulatory approval monitoring tool. The development of practical, affordable assays and the prospective confirmation of prognostic signals in independent, multi-center cohorts are the following crucial stages. Although it has not yet been achieved in ordinary practice, proper integration into treatment pathways—where a biomarker directly influences first-line biologic choice—remains a key objective of PM in RA. It is necessary to move from correlative biomarkers to validated predictive technologies to achieve PM in RA. Although it poses viability issues, synovial pathotyping offers a mechanistic gold standard. The use of blood-based omics markers, especially metabolomics, as readily available substitutes for response prediction and stratification is growing quickly. The next critical step in achieving “the right drug for the right patient” in RA is to incorporate diverse data types into therapeutically actionable algorithms ranked by evidence quality.

## 4 Therapeutic targeting of immunometabolism to treat rheumatoid arthritis

A dysregulated immunometabolic network drives the long-term synovitis and bone loss associated with RA. Inflammation, osteoclast activation, and tissue damage are sustained by the significant metabolic reprogramming that occurs in inflammatory synovial and ICs, which shifts toward glycolysis, changes lipid metabolism, and accumulates immunometabolites such as lactate and succinate. Since over 40% of patients do not react well to current biologic and conventional therapy, this intrinsic dysregulation is a significant cause of treatment resistance (63, 92).

The identification of dysregulated enzymes, transcription factors, metabolites, and whole metabolic pathways in RA synovium has opened a new avenue for potential treatment targets. The local immune response is actively shaped, and chronic inflammation is sustained by factors such as hypoxia, food deprivation, and the buildup of metabolic intermediates. Preclinical research confirms that synovitis can be successfully reduced by targeting specific pathways, such as blocking glycolytic flux or modulating metabolites, including succinate (63, 92, 93).

This thorough mechanistic understanding enables two critical pathways: first, the development of new treatments that directly alter pathogenic metabolism (for example, by using novel pathway inhibitors or repurposed medications such as MET), and second, the tactical use of currently available biologics, guided by metabolic biomarkers that predict response. These new tactics are critically assessed in the following section, with an emphasis on their mechanistic justification and the hierarchy of translational evidence supporting their use in RA.

### 4.1 Metformin: a repurposed metabolic modulator in rheumatoid arthritis

Due to its AMP-activated protein kinase (AMPK)-mediated suppression of critical inflammatory pathways (mTOR, STAT3, HIF-1α), MET, a first-line diabetic medication, is being repurposed for RA and osteoarthritis (OA). Reduced glycolysis, altered T cell differentiation (favoring Tregs over Th17 cells), prevention of osteoclastogenesis, and decreased cytokine production by macrophages and FLS are all consequences of AMPK activation, which regulates mTOR and downstream pro-inflammatory pathways (PI3K/AKT, STAT3, HIF-1α, and NF-κB). Preclinically, it preserves cartilage, reduces synovitis, and alters T-cell balance (by decreasing Th17 and increasing Tregs). Its use has been clinically linked to the reduced incidence and progression of RA/OA, and a preliminary randomized controlled trial (RCT) in RA patients using MTX showed a higher ACR20 response rate. However, before widespread clinical usage can be advised, extensive, conclusive studies are required to validate effectiveness and determine its place in rheumatologic therapy algorithms (94, 95) (**Table 2**).

**Table 2:** Evidence for metformin in rheumatoid arthritis.

Study type	Model/population	Dose/regimen	Primary outcome	Effect size/key finding	Mechanistic readout	Reference
Preclinical *in vitro*	RA FLS, peripheral blood mononuclear cells	1–10 mM	Inhibition of proliferation, cytokine production	Reduced IL-6, TNF-α, MMPs; inhibited FLS invasion	AMPK activation, mTOR inhibition	(94)
Preclinical *in vivo*	CIA mouse model	200–300 mg/kg/day	Arthritis score, histology	Reduced synovitis, cartilage/bone erosion	Increased Tregs, decreased Th17 cells in joints	(94, 95)
RCT	RA patients on MTX	1,000 mg/day + MTX	ACR20 at 12 weeks	80.8% *vs.* 54.7% (placebo), *P* = 0.001	Improved DAS28 remission rates	(96)
RCT	Knee OA, obese patients	Up to 2,000 mg/day	Visual Analog Scale pain at 6 months	-31.3 mm vs. -18.9 mm (placebo)	Reduced serum COMP, C-terminal telopeptide of type I collagen, IL-1β	(97, 98)
Observational cohort	RA/OA patients (database)	N/A (prescription)	Incidence, joint replacement	Associated with a lower risk of RA/OA incidence and progression	N/A	(94)

To treat RA, researchers created MET-derived carbon dots (MCDs), a new nanotherapeutic. MCDs successfully targeted and accumulated in inflamed joints in a rat model of collagen-induced arthritis (CIA), where they decreased fibrosis, synovitis, cartilage degradation, and bone erosion. MCDs scavenge ROS, inhibit the NOD-, LRR-, and pyrin domain-containing protein 3 inflammasome to rewire macrophages from a pro-inflammatory M1 to an anti-inflammatory M2 phenotype, and restore homeostasis in FLSs by blocking the IL-6/gp130 signaling pathway, which, in turn, inhibits their migration, proliferation, and fibrotic activity. MCDs are a viable multi-target treatment approach for RA because of their high biocompatibility (99). Although encouraging, preclinical research and small-to-moderate clinical trials provide the majority of the data supporting MET in RA. To verify effectiveness and determine its position in treatment algorithms, especially in RA populations without diabetes, larger, conclusive phase 3 studies are required.

In 66 RA patients with moderate-to-high disease activity, this prospective, randomized controlled study examined the safety and effectiveness of supplementing standard treatment with MET (850 mg twice daily). Compared with the placebo group, the MET group showed a substantial decrease in both disease activity (DAS28-CRP) and inflammation (CRP) after 6 months. As early as 3 months, the quality of life had considerably improved. Interestingly, whereas serum adiponectin levels rose in the control group, they significantly dropped in the MET group. Except for mild intolerance, which led to a small number of dropouts, the trial found that supplemental MET dramatically reduces inflammation, illness severity, and quality of life in RA patients while maintaining a favorable safety profile (NCT08363405) (100).

In a randomized clinical trial (NCT04068246), adjunctive MET significantly improved treatment outcomes in patients with RA. After 12 weeks, 80.8% of the MET group achieved an ACR20 response compared to 54.7% in the placebo group (*P* = 0.001). The MET group also demonstrated statistically significant and sustained improvements in disease activity (DAS28–3 CRP) from week 4 through week 12, with a significantly higher rate of DAS remission at the study endpoint (*P* = 0.015). Additional benefits were observed in higher response thresholds (ACR50/70) and functional capacity (Health Assessment Questionnaire–Disability Index). The therapy was well tolerated with no significant adverse effects. These results indicate that MET effectively augments the anti-rheumatic action of MTX, presenting a promising adjunct therapy for RA management (101).

Despite promising signs, the use of metformin in the treatment of RA is still under investigation. Though encouraging, the current clinical evidence is based on studies with moderate to small sample sizes. In the absence of large-scale, conclusive phase 3 RCTs in RA-specific populations (especially non-diabetic patients), its effectiveness and ideal placement in treatment algorithms remain uncertain. In addition, while it has a well-established safety profile in diabetics, rheumatological use necessitates close monitoring of gastrointestinal side effects and the extremely rare risk of vitamin B12 deficiency. Despite encouraging preclinical development, clinical testing of targeted formulations such as MCDs is still years away, underscoring the need for innovative delivery methods. To sum up, metformin is an affordable and sensible repurposing option that has strong evidence from both mechanistic and preliminary clinical studies. Strong validation in large-scale prospective trials and a better knowledge of which patient subgroups, perhaps those with particular metabolic or inflammatory markers, would gain the most are prerequisites for its incorporation into routine rheumatologic treatment.

### 4.2 Novel metabolic inhibitor in rheumatoid arthritis

Preclinical research is actively focused on directly pharmacologically suppressing dysregulated metabolic enzymes. These tactics are designed to stop pathogenic cells from getting fuel. Novel inhibitors are being developed to address the unique energy requirements of pathogenic cells in RA, building on the idea of targeting essential metabolic pathways. Dual metabolic inhibition is a crucial tactic that targets the synergistic dysregulation of glutaminolysis and glycolysis seen in hyperactive synovial fibroblasts—for example, c28MS was designed to simultaneously inhibit glutaminase (GLS) in glutaminolysis and HK2 in glycolysis. Co-targeting multiple metabolic requirements is more effective; in preclinical models, c28MS dramatically reduced the severity of arthritis and the aggressiveness of fibroblasts (102, 103).

#### 4.2.1 Glycolysis inhibition

One practical preclinical approach is to directly suppress glycolysis, a characteristic of active immune and stromal cells in RA. By inhibiting pathogenic T- and B-cell responses, 2-deoxy-D-glucose (2-DG), a glycolysis inhibitor, lessens the severity of arthritis in mouse models (such as CIA and K/BxN serum-transfer arthritis). More sophisticated nanodelivery technologies are being investigated to improve selectivity and reduce the systemic toxicity of broad glycolysis inhibition. Dexamethasone-loaded pH/MMP-responsive polymersomes, for example, may target the acidic, protease-rich synovial environment to provide targeted metabolic and anti-inflammatory benefits. These methods are still preclinical; maintaining target-cell selectivity, controlling off-tissue metabolic effects, and demonstrating long-term safety are important translational issues (12, 104). In animal models, 2-DG, a hexokinase inhibitor, has been shown to reduce the severity of arthritis by blocking glycolysis. Advanced delivery techniques have been developed to improve selectivity and lower systemic toxicity—for instance, in animal models, 5-DG and dexamethasone released by stimulus-responsive polymersomes in the acidic, MMP-rich synovial environment have shown better anti-arthritic effects than the free drug. This is a preclinical approach; because glucose is necessary for all healthy tissues, systemic suppression of glycolysis raises serious safety concerns (105, 106).

Through binding to the mitochondrial voltage-dependent anion channel, HK2 plays a crucial role in the glycolytic reprogramming of RA synovial fibroblasts (RASF), increasing their proliferation, invasiveness, and resistance to apoptosis. Because of its limited expression in T cells, targeting HK2 presents a potential, selective therapeutic approach. The small-molecule inhibitor 3-Bromopyruvic Acid (3-BrPA), which reduces arthritis in mice, and methods that target its control via stimulator of interferon genes proteins or m6A methylation are examples of preclinical therapies. Clinically, *HK2* gene polymorphisms are associated with an increased responsiveness to Janus kinase (JAK) inhibitors such as tofacitinib, and 3-BrPA preferentially inhibits FLS glycolysis in patient biopsies. Synovial metabolic activity can now be monitored noninvasively using new methods such as HK2-PET imaging. To improve metabolic PM in RA, future studies should focus on the HK2–lipid metabolism axis and its interaction with biologic therapy (102).

#### 4.2.2 Targeting other pathways

In RA, mtROS are a prospective immunometabolic target and essential signaling molecules. Depending on the particular situation, therapeutically modifying ROS can have conflicting effects—for example, the F1F0-ATPase inhibitor Bz-423 elevates ROS to induce pathogenic lymphocyte apoptosis, thereby selectively protecting lupus models. In contrast, the mtROS scavenger MitoQ reduces excessive ROS production, thereby attenuating pathogenic IFN-γ production. In a similar vein, the enzyme pyruvate dehydrogenase kinase (PDHK)-1 regulates pyruvate entry into the TCA cycle; when it is inhibited, ROS levels rise, promoting the development of Tregs and guarding against autoimmune encephalomyelitis. HIF-1α/HIF-2α, necessary glycolytic enzymes including PFKFB3 and lactate dehydrogenase, which may be regulated to inhibit pathogenic T cell activation and cytokine generation and fundamental drivers of inflammation in the hypoxic joint environment, are other intriguing metabolic targets. The minimal systemic effects observed in preclinical animals suggest that a favorable treatment window may be possible because activated inflammatory cells rely selectively on metabolism, even as systemic suppression of central carbon metabolism raises toxicity concerns (12).

MTX, introduced in the 1980s, remains the cornerstone first-line therapy for RA. Its multifaceted mechanism of action includes inhibiting folate metabolism, altering adenosine signaling, modulating cytokine profiles, and influencing polyamine synthesis, with adenosine-mediated anti-inflammatory effects being a central and widely accepted pathway. While generally effective, long-term MTX use is associated with systemic toxicity, and its precise mechanisms of both efficacy and organ damage, involving pathways such as silent information regulator 1 (SIRT1)/Nrf2 and NF-κB signaling, are still being elucidated. Ongoing research aims to refine MTX’s use by identifying genetic predictors of response and toxicity and optimizing its combination with biologic therapies, such as TNF-α inhibitors (107–109). The safety profile of LD-MTX, a first-line treatment for RA, was clarified in a large RCT (n = 4,786). Over a 23-month follow-up, LD-MTX use was associated with a higher incidence of gastrointestinal, respiratory, viral, and hematologic adverse events, as well as a small increased risk of non-melanoma skin cancer, compared to placebo. Significantly, it did not increase the risk of other cancers or major organ-specific AEs, and it was associated with fewer renal adverse events (110). While MTX remains foundational, JAK inhibitors offer an alternative for MTX-naïve patients. The ORAL Start trial (NCT01039688) demonstrated that tofacitinib monotherapy (5 or 10 mg twice daily) was superior to MTX monotherapy in reducing disease activity (ACR70 response: 25.5%–37.7%, 12.0% at 6 months) and inhibiting radiographic progression in early RA. This efficacy came with a distinct safety profile, including higher rates of herpes zoster infection, increased serum lipids and creatinine, and a noted incidence of malignancies, including lymphoma (111). This underscores the critical need to weigh the superior symptomatic and structural benefits of advanced therapies against their specific risk profiles.

Despite advances in understanding RA pathogenesis and the development of targeted biologics, including TNF and IL-6 inhibitors and JAK inhibitors, a significant proportion of patients experience inadequate responses to conventional DMARDs, corticosteroids, and non-steroidal anti-inflammatory drugs. This unmet need is driving innovation beyond traditional small molecules toward emerging modalities, including cell-based therapies, RNA-targeted treatments, and phytocannabinoids, which represent the next frontier in the pursuit of more effective and personalized RA management (109, 112, 113).

Rapid increases in glucose absorption and glycolysis are essential for IC activation and adaptability, according to research in IM. GLUT1, which is highly increased in activated T cells, B cells, and macrophages, is essential to this metabolic transition. Its inhibition reduces inflammatory responses, effector functions, and proliferation. This glycolytic hyperactivity characterizes autoimmunity; in RA, the hypoxic synovial milieu increases GLUT1 expression and lactate production in FLSs and infiltrating ICs. Significantly, in mouse models of psoriasis, lupus, and RA, genetic or pharmacological reduction of GLUT1/glycolysis reduces illness, confirming the therapeutic targeting of immunometabolic pathways in autoimmune disorders (114).

The Warburg effect, accelerated glycolysis with suppressed OXPHOS, depletes NADH pools, leading cells to compensate via glutaminolysis. While underexplored in arthritis, GLS-1 inhibition has been shown to suppress RA fibroblast (FLS) proliferation and to ameliorate disease in SKG mice. Furthermore, Th17 cells exhibit heightened FAS expression, and blocking this pathway with soraphen A inhibits Th17 differentiation, promotes Treg expansion, and alleviates experimental autoimmune encephalomyelitis. Although FAS does not directly produce ATP or NADH, its inhibition likely disrupts membrane lipid biosynthesis, which is essential for Th17 effector functions. These findings underscore the therapeutic potential of targeting compensatory metabolic pathways beyond glycolysis. Consequently, metabolism-modifying nutritional interventions are now entering clinical evaluation for rheumatic diseases (ClinicalTrials.gov NCT02941055) (115, 116).

By focusing on hypermetabolic synovial fibroblasts, researchers assessed the combined inhibition of glycolysis and glutaminolysis as a treatment approach for RA. A subset of pathogenic fibroblasts co-expresses the glutaminolytic enzyme GLS, the glycolytic enzyme HK2, and pro-inflammatory genes, according to scRNA-seq. *In vitro*, c28MS, a new phytobiological molecule, concurrently inhibited both pathways, reducing the aggressive phenotype of RA fibroblasts and their production of IL-6, CCL2, and MMP3. Administration of c28MS significantly reduced arthritis severity in the K/BxN mouse model. According to these results, RA and other non-cancer disorders caused by metabolic reprogramming may be effectively treated by co-targeting glucose and glutamine metabolism (117).

Researchers discovered critical glycolytic enzymes that are dysregulated in the pathophysiology of RA. The glycolytic enzymes Enolase 1, HK2, and phosphoglycerate kinase 1 (PGK1) were increased in ST from clinical RA patients and in a CIA model, whereas the gluconeogenic genes phosphoenolpyruvate carboxykinase 1 and PDHK-4 were downregulated. Both patient blood and RA synovium had significantly higher levels of the enzyme PGK1. In RASF, functional validation demonstrated that silencing PGK1 with siRNA markedly decreased cell migration, proliferation, and the synthesis of pro-inflammatory cytokines IFN-γ and IL-1β. These results establish PGK1 as a new biomarker and potential target for metabolic therapy in RA, linked to synovial inflammation and hyperplasia (118).

Animal models of arthritis have shown that many metabolism inhibitors can help treat the condition. Several enzymes that slow glycolysis have been suggested as potential therapeutic targets. Researchers have already found that blocking HK2 with 3-BrPA can convert Th17 cells into Treg cells after *in vitro* stimulation (Th17 conditions: IL6, TGF-β, anti-IL4 Ab, anti-IFN-γ Ab, IL-2) and improve arthritis in SKG mice. Panneton et al. also showed that BrPA reduced disease activity in a CIA model. Bian et al. found that blocking PDK with dichloroacetate also helped CIA mice with arthritis (116, 119, 120).

### 4.3 Natural compounds with defined immunometabolic targets

These new inhibitors are mainly in the preclinical stage despite their potential. Achieving cell-type selectivity (i.e., targeting fibroblasts over ICs), ensuring a sufficient therapeutic window to prevent systemic metabolic toxicity, and developing effective delivery mechanisms to the synovium are crucial translation challenges. To identify individuals with malignancies or STs that display the particular metabolic vulnerabilities that these medications are intended to exploit, future clinical use will likely rely on integration with biomarker methods. Achieving cell-type- or tissue-specific targeting to prevent global metabolic disruption is the main translational challenge for new metabolic inhibitors. Future research must focus on prodrug methods and nanotechnology triggered by the inflammatory microenvironment (121).

Traditional Chinese medicine (TCM), with its long history of treating refractory conditions such as RA, offers a rich source of bioactive compounds whose mechanisms are now being elucidated using omics technologies—for example, the approved TCM-derived alkaloid sinomenine exerts its anti-arthritic effect by specifically inhibiting the prostaglandin E2-producing enzyme microsomal prostaglandin E synthase-1. Emerging evidence suggests that the anti-inflammatory and immunomodulatory benefits of many TCM compounds are mediated through the targeted regulation of immunometabolic pathways, offering a novel, mechanism-based rationale for their therapeutic application in RA (122).

A naturally occurring alkaloid, berberine, has strong anti-arthritic properties by modulating immunometabolic pathways through multiple targets. By blocking essential pathways, including AMPK/lipogenesis, lysophosphatidic acid/extracellular signal-regulated kinase/p38 MAPK signaling, and ROS-AMPK, it inhibits the inflammatory proliferation of RAFLS. It also lowers palmitic acid levels by downregulating the lipid metabolism regulator sterol regulatory element-binding protein 1. Additionally, berberine reverses the pro-glycolytic shift in CD4+ T cells and suppresses mTORC1/HIF-1α signaling in M1 macrophages by activating AMPK, which counteracts glycolytic reprogramming. Notably, berberine has a good safety profile at clinical doses without causing cytotoxicity or mutagenicity. It also has the potential to work in concert with diclofenac sodium to lessen intestinal mucosal injury. Berberine is a potential multi-pathway metabolic modulator for RA treatment because of these characteristics (26, 123, 124).

Because it activates SIRT1, resveratrol, a naturally occurring phytohormone, is a viable treatment option for RA. This activation triggers a wide-ranging regulatory cascade that suppresses critical inflammatory pathways, including NF-κB and MAPK, leading to decreased production of MMPs and pro-inflammatory cytokines, such as interleukins. At the cellular level, resveratrol controls macrophage polarization, preserves mitochondrial redox balance, reduces synovial inflammation, hyperplasia, and cartilage degradation, and triggers apoptosis in hyperactive synovial fibroblasts (such as MH7A cells) and inflammatory cells. Additionally, it could lessen comorbidities such as interstitial pneumonia and periodontitis that are linked to RA. Resveratrol’s clinical translation is still pending validation through large-scale, multi-center trials and the development of sophisticated delivery systems (such as targeted, controlled-release formulations) to overcome its pharmacokinetic limitations and demonstrate its effectiveness as a next-generation anti-RA agent despite strong preclinical evidence (125).

Natural compounds have a long history of use, but they often suffer from poor bioavailability, lack of standardization, and a scarcity of rigorous, large-scale RCTs meeting modern drug development standards. Despite compelling preclinical results, this entire class of agents faces a formidable translational challenge: achieving a sufficient therapeutic window. Off-target toxicity is a significant risk when systemically inhibiting central metabolic pathways, such as glycolysis, as these processes are fundamental to all cells, especially in highly metabolically active tissues.

### 4.4 Using biomarkers to guide existing biological and targeted therapies

The more intelligent, biomarker-guided use of current treatments is the most direct therapeutic use of immunometabolic findings. By matching patients with the best medication based on molecular characteristics, this method goes beyond trial-and-error. Stratifying patients by synovial B-cell molecular signatures is the most obvious example; the R4RA study showed that individuals without this signature are unlikely to respond to rituximab, making tocilizumab (anti-IL-6R) a more sensible option. On the other hand, a myeloid/glycolytic signature, characterized by elevated lactate, succinate, and glycolytic enzymes, suggests that the IL-6/JAK/STAT3 axis is dominant. This indicates that JAK inhibitors or IL-6 receptor antagonists should be given priority. Additionally, integrating metabolic management, such as lifestyle modifications and MET, with immunosuppression to improve response and reduce cardiovascular risk is supported by recognition that systemic metabolic dysregulation, such as insulin resistance, is a cause of poor outcomes (29, 73, 112, 113, 126).

Even with the wide range of RA treatments available, such as standard DMARDs, TNF inhibitors, IL-6R blockers, B-cell depleting drugs, and JAK inhibitors, a significant percentage of patients still do not experience long-lasting remission. Thus, biomarker-guided selection of existing immunomodulators and the selective incorporation of direct metabolic-modulating drugs are key components of the future of RA treatment. The next frontier in PM for RA is a dual approach that uses proven biomarkers to tailor current medications and develop new metabolic therapies (112, 113, 126).

Ultimately, there are two main avenues for RA IM therapy: first, the biomarker-guided strategic deployment of existing biologics; second, the innovative but risky development of novel metabolic inhibitors and repurposed drugs. The latter faces substantial challenges regarding specificity and safety, but if successful, it could lead to mechanistic treatments that reset pathogenic cellular metabolism. The future of RA treatment lies in integrating these two approaches, guided by precision diagnostics, to achieve deep, sustained remission.

## 5 Personalized medicine approaches in rheumatoid arthritis: a critical appraisal

RA is an excellent target for PM due to its genetic heterogeneity, clinical presentation, and treatment response. This method goes beyond a general “trial-and-error” approach by using biomarkers to stratify patients and forecast the most successful course of therapy. High-resolution techniques, such as single-cell and spatial transcriptomics, which have identified unique pathogenic cell subsets within the synovium, and multi-omics integration, which connects these cellular profiles to clinical outcomes, are driving progress toward PM (127–129).

### 5.1 Validated biomarker-driven therapeutic stratification

The most sophisticated PM strategy for RA uses ST biomarkers to guide biologic selection. A fundamental, yet complex, example of this approach is the R4RA (rituximab vs. tocilizumab in anti-TNF inadequate responder) study (29, 74). Patients with a molecularly defined B-cell-poor synovial signature responded significantly better to tocilizumab than to rituximab, according to a subsequent RNA sequencing re-analysis, even though the initial histological stratification did not indicate a significant difference in clinical response. This result underscores a crucial idea: the accuracy of the molecular assay determines the assay’s predictive power. The absolute response rates (e.g., 50% vs. 12%) should be interpreted in light of the fact that the R4RA cohort comprised anti-TNF inadequate responders, a specific patient population. Although molecular pathotyping does not constitute a universal, deterministic rule, the data strongly suggest that it can meaningfully inform drug selection, going beyond serology. The pauci-immune/fibroid pathotype also emerged as a possible marker of broader drug resistance, underscoring the intricacy of synovial biology. Before these encouraging findings can be translated into general-use treatment algorithms, they must be validated in larger, independent, and more varied cohorts, along with the development of standardized, clinically viable assays (75).

Biomarkers in RA serve distinct clinical purposes: predictive biomarkers, such as synovial transcriptomic clusters encompassing myeloid versus lymphoid profiles, are emerging tools to forecast differential responses to specific biologic therapies (37, 86); prognostic or monitoring biomarkers, exemplified by the validated MBDA score, quantify disease activity and correlate with long-term outcomes but do not yet inform initial treatment decisions (87); and associative biomarkers, which include most current metabolite signatures, such as specific lipids or TCA cycle intermediates, primarily reflect disease state or activity. However, early metabolomic panels show promising potential for predicting treatment response to agents such as MTX (109, 112, 113, 130).

### 5.2 Patient stratification based on metabolic and systemic profiles

Systemic metabolic profiles provide an additional stratification axis beyond ST. The necessity for integrated cardio-metabolic therapy is highlighted by the poorer outcomes and increased disease activity seen in patients with RA and co-existing metabolic syndrome (131). Specific plasma lipid and energy metabolites, such as ceramides and sphingomyelins, are linked to the MTX response, according to pharmacometabolomic research (132). Nevertheless, a machine learning study using blood lipid profiles was unable to predict MTX response at 6 months with greater accuracy than clinical variables, suggesting that not all metabolic markers can be translated immediately (33).

### 5.3 Integration of multi-omics data and future directions

Deciphering the intricacies of RA requires integrating genomic, transcriptomic, proteomic, and metabolic data, aided by artificial intelligence. To identify syndrome-specific biomarkers, this multi-omics method has also been used to construct TCM syndromes (e.g., RA-cold vs. RA-hot) (133). To develop strong algorithms that can guide first-line therapy, enhance outcomes, and reduce the socioeconomic burden of ineffective treatment, PM in RA must be prospectively validated in large, diverse cohorts and integrated with clinical data.

A clear transition from correlative discovery to interventional validation via prospective, biomarker-stratified studies that demonstrate clinical value is essential for the road ahead in personalized treatment in RA. To capture the dynamic evolution of disease biology, this must be combined with the development of practical, standardized, and affordable tests suitable for routine treatment and longitudinal investigations. Furthermore, to ensure fair application, robust validation across large, heterogeneous, real-world populations is necessary. In the end, the next 10 years must be devoted to closing the enormous translational gap between the development of useful, point-of-care technologies that clearly enhance patient outcomes and high-resolution molecular knowledge.

## 6 Future perspectives and translational challenges

Although many obstacles remain, the application of immunometabolic findings to RA clinical practice is encouraging. Achieving safety and specificity is a significant task. To attain synovial specificity, future techniques rely on targeted delivery platforms (such as pH- or MMP-responsive nanocarriers); systemic suppression of basic processes, such as glycolysis, poses off-target risks. Additionally, most metabolic biomarkers remain in the discovery stage despite their promise. To prove clinical value and standardize tests, their clinical translation needs prospective validation in large, multicenter cohorts (134). To create predictive models of patient-specific cellular ecosystems and metabolic relationships, single-cell and spatial omics technologies must be integrated with artificial intelligence, given the extreme variability of the human RA synovium. Novel immunotherapeutic targets may become apparent as our understanding of the metabolic regulation of DCs, which undergo metabolic reprogramming in the hypoxic synovium, and of Tregs, whose activity is dysregulated in RA, deepens (135–140).

Additionally, the development of cell- or tissue-specific delivery methods is required, as systemic inhibition of essential processes, such as glycolysis, can cause off-target damage, underscoring the importance of specificity and safety. Furthermore, biomarker validation is necessary; large, prospective studies are needed to establish clinical utility for the majority of synovial pathotypes and omics signatures, which are currently in the discovery/validation stages. Artificial intelligence and multi-omics data integration may help with this. To characterize the intricate metabolic interactions within the human RA synovium and capture patient-specific cellular ecosystems, single-cell and spatial omics methods must go beyond animal models and small cohorts to address human variability (47, 141–146). The absence of practical, standardized tests that can be integrated into routine clinical workflows to guide treatment at the point of care is a crucial gap. Additionally, our knowledge is primarily correlative; stronger evidence is needed to establish causal relationships between specific metabolic changes and long-term treatment outcomes (147–149).

Novel treatment approaches include investigating dietary and microbiota interventions as supplemental immunometabolic therapy as well as directly manipulating key signaling metabolites such as lactate and succinate. Crucially, the research needs to confirm results in human trials by going beyond small cohort studies and animal models. Pre-RA intervention, which uses metabolic and serologic markers to identify at-risk patients for trials with metabolic modulators (such as MET) to postpone or avoid clinical onset, is a primary translational objective. Finally, metabolic reprogramming drugs combined with biomarker-guided biologic treatment may provide a way to reset the underlying immunological malfunction and achieve long-lasting remission (150–154).

**Figure 4:**
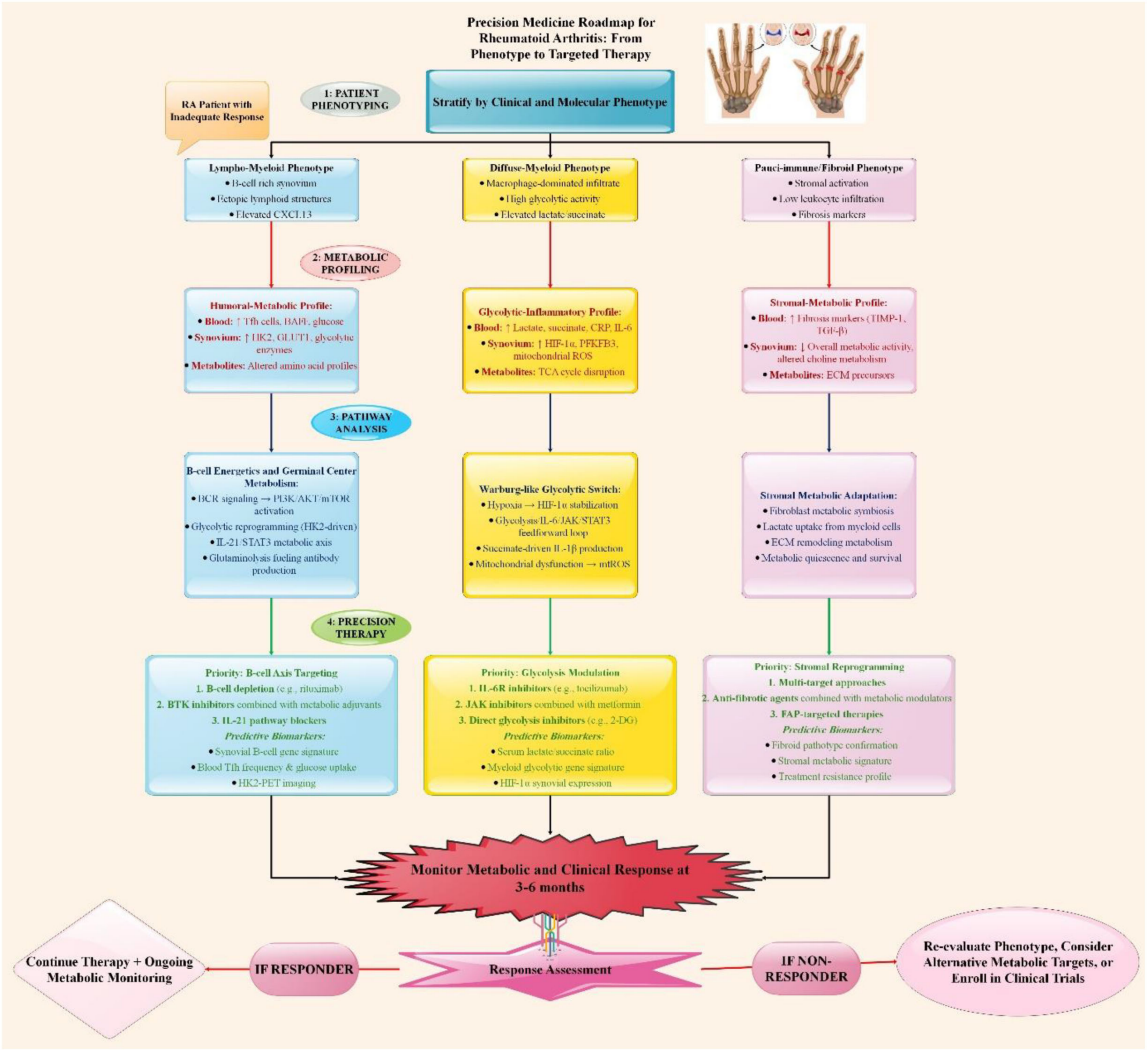
A path for immunometabolic precision therapy in RA. This decision algorithm describes a four-step paradigm for classifying patients with active RA and directing targeted treatment selection based on the underlying immunometabolic disease. (1) Patient phenotyping: Based on systemic biomarkers and synovial histology, patients are categorized into one of three molecular phenotypes. (2) Metabolic profiling: Using blood-based tests like metabolomics and synovial readouts, which include gene expression and imaging, each phenotype is linked to a unique immunometabolic profile. (3) Pathway analysis: The predominant dysregulated immunometabolic circuits causing illness in each subgroup are shown by these metabolic profiles. (4) Precision therapy: Using predictive biomarkers to predict response, the revealed pathways guide the selection of treatment classes that are prioritized. Every patient has a response review every 3 to 6 months; non-responders begin a reevaluation cycle. Metabolic indicators are essential tools for transitioning from empirical to precision-based therapy of RA because the integrated model highlights that metabolic reprogramming is a primary pathogenic driver rather than just a bystander effect.

Closing these gaps will be key to the future of PM in RA. The way ahead necessitates (1) using multi-omics (single-cell transcriptomics, spatial metabolomics) and deep phenotyping on well-characterized patient cohorts to identify illness endotypes and their underlying metabolic mechanisms, (2) going beyond retrospective correlation and thoroughly confirming predictive biomarkers in interventional studies that evaluate biomarker-stratified treatment approaches, (3) creating and implementing intelligent clinical technologies that provide physicians with actionable findings, including minimally invasive synovial profiling or integrated blood-based tests, (4) adopting a comprehensive PM framework that creates multi-modal prediction models by integrating immunometabolic profiling with genetic, microbiome, and lifestyle data. The creation of workable algorithms that consistently match patients to the best treatment—whether a targeted biologic, a novel pathway inhibitor, or a repurposed metabolic drug—at the earliest stage will ultimately determine success. This will change the management of RA from iterative suppression to customized interception and the restoration of immune-metabolic balance (**Figure 4**).

## 7 Conclusion

By demonstrating how cell-type-specific metabolic reprogramming in T cells, macrophages, B cells, and synovial fibroblasts directly feeds synovitis, autoimmunity, and joint destruction, IM offers a revolutionary paradigm to comprehend RA. A new age of PM in RA is based on this mechanistic understanding. Instead of being a single illness, RA comprises multiple molecular endotypes, including diffuse myeloid, pauci-immune/fibroid, and lympho-myeloid synovial pathotypes, each influenced by distinct metabolic and inflammatory circuits.

The most immediate clinical application of this knowledge is the more innovative, biomarker-guided use of existing therapies. Synovial B-cell signatures can predict response to B-cell depletion, while myeloid/glycolytic profiles indicate susceptibility to IL-6 or JAK inhibition, moving treatment selection beyond trial and error. Concurrently, the therapeutic pipeline is expanding to include novel agents that directly target pathogenic metabolism, such as glycolysis inhibitors, modulators of key immunometabolites, including succinate, lactate, and repurposed drugs like MET. However, significant translational challenges remain. These include ensuring the specificity and safety of metabolic inhibitors, prospectively validating predictive biomarkers in large, diverse cohorts, and deciphering the profound heterogeneity of the human synovium through integrated multi-omics and spatial technologies. Combining these two approaches—developing and incorporating new metabolic modulators and using proven molecular markers to tailor the selection of existing biologic and targeted therapies—will be key to managing RA in the future. By combining sophisticated analytics with a thorough understanding of immunometabolic interactions, this approach has the potential to achieve long-lasting remission and eventually help prevent this crippling illness.

## Glossary

ACPA: anti-citrullinated protein antibody
AMPK: AMP-activated protein kinase
AUC: area under the curve
BAFF: B-cell activating factor
bDMARDs: biologic disease-modifying antirheumatic drugs
3-BrPA: 3-bromopyruvic acid
BTK: Bruton’s tyrosine kinase
CCP: cyclic citrullinated peptide
cDCs: conventional dendritic cells
CIA: collagen-induced arthritis
CRP: C-reactive protein
CTX-1: C-terminal telopeptide of type I collagen
cTfh: circulating T follicular helper
DAMPs: damage-associated molecular patterns
DAS28: disease activity score (28 joints)
DCs: dendritic cells
DMARDs: disease-modifying antirheumatic drugs
ECM: extracellular matrix
ENO1: enolase 1
FAS: fatty acid synthesis
FAO: fatty acid oxidation
FDA: food and drug administration
FGF-21: fibroblast growth factor 21
FLS: fibroblast-like synoviocytes
GLUT1: glucose transporter 1
GLS: glutaminase
HIF-1α: hypoxia-inducible factor 1-alpha
HK2: hexokinase 2
IC: immune cell
IFN-γ: interferon gamma
IL: Interleukin
IM: Immunometabolism
JAK: Janus kinase
LD: low-dose
MAPK: mitogen-activated protein kinase
MBDA: multi-biomarker disease activity
MCDs: metformin-derived carbon dots
MET: metformin
MMP: matrix metalloproteinase
moDCs: monocyte-derived dendritic cells
mtROS: mitochondrial reactive oxygen species
mTOR: mechanistic target of rapamycin
MTX: methotrexate
NIRRA: non-inflammatory refractory RA
NLRP3: NOD-, LRR-, and pyrin domain-containing protein 3
NMR: nuclear magnetic resonance
Nrf2: nuclear factor erythroid 2-related factor 2
OA: osteoarthritis
OXPHOS: oxidative phosphorylation
pDCs: plasmacytoid dendritic cells
PFKFB3: 6-phosphofructo-2-kinase/fructose-2,6-biphosphatase 3
PGK1: phosphoglycerate kinase 1
PI3K: phosphoinositide 3-kinase
PIRRA: persistent inflammatory refractory rheumatoid arthritis
PM: precision medicine
RA: rheumatoid arthritis
RANKL: receptor activator of nuclear factor kappa-B ligand
RASF: RA synovial fibroblasts
RCT: randomized controlled trial
RF: rheumatoid factor
RNA-seq: RNA Sequencing
ROS: reactive oxygen species
scRNA-seq: single-cell RNA sequencing
SIRT1: sirtuin 1
STAT3: signal transducer and activator of transcription 3
ST: synovial tissue
TCA: tricarboxylic acid
TCM: traditional Chinese medicine
Tfh: T follicular helper
Th: T helper cell
TNF: tumor necrosis factor
Tph: peripheral helper T cell
Treg: regulatory T cell
US: ultrasound

## References

[1] Thorp EB Karlstaedt A . Intersection of immunology and metabolism in myocardial disease. Circ Res. (2024) 134:1824–40. doi: 10.1161/CIRCRESAHA.124.323660, PMID: 38843291 PMC11569846

[2] Tan W Pan T Wang S Li P Men Y Tan R . Immunometabolism modulation, a new trick of edible and medicinal plants in cancer treatment. Food Chem. (2022) 376:131860. doi: 10.1016/j.foodchem.2021.131860, PMID: 34971892

[3] Paolicelli RC Angiari S . Microglia immunometabolism: From metabolic disorders to single cell metabolism. Semin Cell Dev Biol. (2019) 94:129–37. doi: 10.1016/j.semcdb.2019.03.012, PMID: 30954657

[4] Cui Y Feng Z Zhao Q Dai H Zheng Y Rui H-L . Immunocyte lipid metabolic reprogramming: a novel pathway for targeted intervention in autoimmune diseases. Front Immunol. (2025) 16:1713148. doi: 10.3389/fimmu.2025.1713148, PMID: 41280910 PMC12631377

[5] Pająk B Zieliński R Priebe W . The impact of glycolysis and its inhibitors on the immune response to inflammation and autoimmunity. Molecules. (2024) 29:1298. doi: 10.3390/molecules29061298, PMID: 38542934 PMC10975218

[6] Castello A Hentze MW Preiss T . Metabolic enzymes enjoying new partnerships as RNA-binding proteins. Trends Endocrinol Metab. (2015) 26:746–57. doi: 10.1016/j.tem.2015.09.012, PMID: 26520658 PMC4671484

[7] Hotamisligil GS . Foundations of immunometabolism and implications for metabolic health and disease. Immunity. (2017) 47:406–20. doi: 10.1016/j.immuni.2017.08.009, PMID: 28930657 PMC5627521

[8] Hu T Liu C-H Lei M Zeng Q Li L Tang H . Metabolic regulation of the immune system in health and diseases: mechanisms and interventions. Signal Transduction Targeted Ther. (2024) 9:268. doi: 10.1038/s41392-024-01954-6, PMID: 39379377 PMC11461632

[9] Alshammary RA Khadim MM Al-Karawi AS Kadhim AS Ahmed HY Laftah AR . Biomarkers of sepsis severity: A comparative evaluation of immunological and biochemical parameters. Al-Anbar Med J. (2025) 21:255–62. doi: 10.33091/amj.2025.159680.2222

[10] Al-Karawi AS Kadhim AS . Correlation of autoimmune response and immune system components in the progression of IgA nephropathy: A comparative study. Hum Immunol. (2024) 85:111181. doi: 10.1016/j.humimm.2024.111181, PMID: 39566436

[11] Kadhim AS Al-Karawi AS . Investigating the multifactorial correlation between obesity and rheumatoid arthritis: a study of immunological and biochemical markers. Obes Med. (2025) 53:100578. doi: 10.1016/j.obmed.2024.100578, PMID: 41774992

[12] Rhoads JP Major AS Rathmell JC . Fine tuning of immunometabolism for the treatment of rheumatic diseases. Nat Rev Rheumatol. (2017) 13:313–20. doi: 10.1038/nrrheum.2017.54, PMID: 28381829 PMC5502208

[13] Golstein MA . Diagnostic errors in rheumatology and medico-legal consequences. Medico-Legal J. (2024) 92:139–44. doi: 10.1177/00258172241235016, PMID: 38757615

[14] Kłak A Raciborski F Samel-Kowalik P . Social implications of rheumatic diseases. Reumatologia/Rheumatology. (2016) 54:73–8. doi: 10.5114/reum.2016.60216, PMID: 27407283 PMC4918047

[15] Hanlon MM Canavan M Barker BE Fearon U . Metabolites as drivers and targets in rheumatoid arthritis. Clin Exp Immunol. (2022) 208:167–80. doi: 10.1093/cei/uxab021, PMID: 35020864 PMC9188347

[16] Zheng J Yin J Huang R Petersen F Yu X . Meta-analysis reveals an association of STAT4 polymorphisms with systemic autoimmune disorders and anti-dsDNA antibody. Hum Immunol. (2013) 74:986–92. doi: 10.1016/j.humimm.2013.04.034, PMID: 23628400

[17] Canavan M Marzaioli V Mcgarry T Bhargava V Nagpal S Veale D . Rheumatoid arthritis synovial microenvironment induces metabolic and functional adaptations in dendritic cells. Clin Exp Immunol. (2020) 202:226–38. doi: 10.1111/cei.13479, PMID: 32557565 PMC7597596

[18] Zhao F Hu Z Li G Liu M Huang Q Ai K . Angiogenesis in rheumatoid Arthritis: Pathological characterization, pathogenic mechanisms, and nano-targeted therapeutic strategies. Bioactive Materials. (2025) 50:603–39. doi: 10.1016/j.bioactmat.2025.04.026, PMID: 40453697 PMC12124647

[19] Chang X Wei C . Glycolysis and rheumatoid arthritis. Int J rheumatic Dis. (2011) 14:217–22. doi: 10.1111/j.1756-185X.2011.01598.x, PMID: 21816017

[20] Yue S Fan J Xie D Cao C Wang Z Huang J . Unveiling the therapeutic potential: targeting fibroblast-like synoviocytes in rheumatoid arthritis. Expert Rev Mol Med. (2025) 27:1–24. doi: 10.1017/erm.2025.11, PMID: 40468839 PMC12201960

[21] Weyand CM Goronzy JJ . Metabolic checkpoints in rheumatoid arthritis. Semin Arthritis Rheumatism. (2025) 70:152586. doi: 10.1016/j.semarthrit.2024.152586, PMID: 39550308 PMC11761375

[22] Jia Y Li R Huang L Wu X Zhao L Yang H . The Glycolysis-HIF-1α axis induces IL-1β of macrophages in rheumatoid arthritis. Arthritis Res Ther. (2025) 27:180. doi: 10.1186/s13075-025-03647-z, PMID: 41013704 PMC12465665

[23] Szabó MZ Szodoray P Kiss E . Dyslipidemia in systemic lupus erythematosus. Immunologic Res. (2017) 65:543–50. doi: 10.1007/s12026-016-8892-9, PMID: 28168401

[24] Zhang J Fang X-Y Leng R Chen H-F Qian T-T Cai Y-Y . Metabolic signature of healthy lifestyle and risk of rheumatoid arthritis: observational and Mendelian randomization study. Am J Clin Nutr. (2023) 118:183–93. doi: 10.1016/j.ajcnut.2023.04.034, PMID: 37127109

[25] Cao S Li Y Song R Meng X Fuchs M Liang C . L-arginine metabolism inhibits arthritis and inflammatory bone loss. Ann rheumatic Dis. (2024) 83:72–87. doi: 10.1136/ard-2022-223626, PMID: 37775153 PMC10803985

[26] Hu S Lin Y Tang Y Zhang J He Y Li G . Targeting dysregulated intracellular immunometabolism within synovial microenvironment in rheumatoid arthritis with natural products. Front Pharmacol. (2024) 15:1403823. doi: 10.3389/fphar.2024.1403823, PMID: 39104392 PMC11298361

[27] Thomas J Bansback N Barber C Wells G Hazlewood G . Personalized medicine in rheumatoid arthritis: Combining biomarkers and patient preferences to guide therapeutic decisions. Best Pract Res Clin Rheumatol. (2022) 36:101812. doi: 10.1016/j.berh.2022.101812, PMID: 36653230

[28] Sebastiani M Vacchi C Manfredi A Cassone G . Personalized medicine and machine learning: a roadmap for the future. MDPI J. Clin. Med. (2022) Available online at: https://www.mdpi.com/2077-0383/11/14/4110 (Accesed July 9, 2025). 10.3390/jcm11144110 PMC9317385 35887873

[29] Rivellese F Surace AE Goldmann K Sciacca E Çubuk C Giorli G . Rituximab versus tocilizumab in rheumatoid arthritis: synovial biopsy-based biomarker analysis of the phase 4 R4RA randomized trial. Nat Med. (2022) 28:1256–68. doi: 10.1038/s41591-022-01789-0, PMID: 35589854 PMC9205785

[30] De Jong TD Vosslamber S Verweij CL . Moving towards personalized medicine in rheumatoid arthritis. Springer Arthritis Res. Ther. (2014) Available online at: https://link.springer.com/article/10.1186/ar4565 (Accesed July 9, 2025). 10.1186/ar4565 PMC4060201 25166016

[31] Badii M Gaal O Popp RA Crişan TO Joosten LA . Trained immunity and inflammation in rheumatic diseases. Joint Bone Spine. (2022) 89:105364. doi: 10.1016/j.jbspin.2022.105364, PMID: 35219890

[32] Xu L Chang C Jiang P Wei K Zhang R Jin Y . Metabolomics in rheumatoid arthritis: Advances and review. Front Immunol. (2022) 13:961708. doi: 10.3389/fimmu.2022.961708, PMID: 36032122 PMC9404373

[33] Maciejewski M Sands C Nair N Ling S Verstappen S Hyrich K . Prediction of response of methotrexate in patients with rheumatoid arthritis using serum lipidomics. Sci Rep. (2021) 11:7266. doi: 10.1038/s41598-021-86729-7, PMID: 33790392 PMC8012618

[34] Blackmore D Li L Wang N Maksymowych W Yacyshyn E Siddiqi ZA . Metabolomic profile overlap in prototypical autoimmune humoral disease: a comparison of myasthenia gravis and rheumatoid arthritis. Metabolomics. (2020) 16:1–15. doi: 10.1007/s11306-019-1625-z, PMID: 31902059

[35] Cui L Weiyao J Chenghong S Limei L Xinghua Z Bo Y . Rheumatoid arthritis and mitochondrial homeostasis: The crossroads of metabolism and immunity. Front Med. (2022) 9:1017650. doi: 10.3389/fmed.2022.1017650, PMID: 36213670 PMC9542797

[36] Bustamante MF Oliveira PG Garcia-Carbonell R Croft AP Smith JM Serrano RL . Hexokinase 2 as a novel selective metabolic target for rheumatoid arthritis. Ann rheumatic Dis. (2018) 77:1636–43. doi: 10.1136/annrheumdis-2018-213103, PMID: 30061164 PMC6328432

[37] Qiu J Wu B Goodman SB Berry GJ Goronzy JJ Weyand CM . Metabolic control of autoimmunity and tissue inflammation in rheumatoid arthritis. Front Immunol. (2021) 12:652771. doi: 10.3389/fimmu.2021.652771, PMID: 33868292 PMC8050350

[38] Fearon U Hanlon MM Floudas A Veale DJ . Cellular metabolic adaptations in rheumatoid arthritis and their therapeutic implications. Nat Rev Rheumatol. (2022) 18:398–414. doi: 10.1038/s41584-022-00771-x, PMID: 35440762

[39] Hitchon CA El-Gabalawy HS . Oxidation in rheumatoid arthritis. Arthritis Res Ther. (2004) 6:265–78. doi: 10.1186/ar1447, PMID: 15535839 PMC1064874

[40] Vaamonde-García C López-Armada MJ . Role of mitochondrial dysfunction on rheumatic diseases. Biochem Pharmacol. (2019) 165:181–95. doi: 10.1016/j.bcp.2019.03.008, PMID: 30862506

[41] Zheng Y Wei K Jiang P Zhao J Shan Y Shi Y . Macrophage polarization in rheumatoid arthritis: signaling pathways, metabolic reprogramming, and crosstalk with synovial fibroblasts. Front Immunol. (2024) 15:1394108. doi: 10.3389/fimmu.2024.1394108, PMID: 38799455 PMC11116671

[42] Cen S Wang P Xie Z Yang R Li J Liu Z . Autophagy enhances mesenchymal stem cell-mediated CD4+ T cell migration and differentiation through CXCL8 and TGF-β1. Stem Cell Res Ther. (2019) 10:1–13. doi: 10.1186/s13287-019-1380-0, PMID: 31443687 PMC6708254

[43] Stathopoulou C Nikoleri D Bertsias G . Immunometabolism: an overview and therapeutic prospects in autoimmune diseases. Immunotherapy. (2019) 11:813–29. doi: 10.2217/imt-2019-0002, PMID: 31120393

[44] Rao DA Gurish MF Marshall JL Slowikowski K Fonseka CY Liu Y . Pathologically expanded peripheral T helper cell subset drives B cells in rheumatoid arthritis. Nature. (2017) 542:110–4. doi: 10.1038/nature20810, PMID: 28150777 PMC5349321

[45] Xu X Zhou J Xie H Zhang R Gu B Liu L . Immunomodulatory mechanisms of the gut microbiota and metabolites on regulatory T cells in rheumatoid arthritis. Front Immunol. (2025) 16:1610254. doi: 10.3389/fimmu.2025.1610254, PMID: 40692793 PMC12277365

[46] Chin A Small A Wong SW Wechalekar MD . T cell dysregulation in Rheumatoid Arthritis: from genetic susceptibility to established disease. Curr Rheumatol Rep. (2025) 27:14. doi: 10.1007/s11926-025-01180-1, PMID: 39862300 PMC11762599

[47] Gao A Wu R Mu Y Jin R Jiang S Gao C . Restoring immune tolerance in pre-RA: immunometabolic dialogue between gut microbiota and regulatory T cells. Front Immunol. (2025) 16:1565133. doi: 10.3389/fimmu.2025.1565133, PMID: 40181974 PMC11965651

[48] Mondal S Saha S Sur D . Immuno-metabolic reprogramming of T cell: a new frontier for pharmacotherapy of Rheumatoid arthritis. Immunopharmacol Immunotoxicology. (2024) 46:330–40. doi: 10.1080/08923973.2024.2330636, PMID: 38478467

[49] Roberts CA Dickinson AK Taams LS . The interplay between monocytes/macrophages and CD4+ T cell subsets in rheumatoid arthritis. Front Immunol. (2015) 6:571. doi: 10.3389/fimmu.2015.00571, PMID: 26635790 PMC4652039

[50] Salnikova DI Nikiforov NG Postnov AY Orekhov AN . Target role of monocytes as key cells of innate immunity in rheumatoid arthritis. Diseases. (2024) 12:81. doi: 10.3390/diseases12050081, PMID: 38785736 PMC11119903

[51] Shirai T Nazarewicz RR Wallis BB Yanes RE Watanabe R Hilhorst M . The glycolytic enzyme PKM2 bridges metabolic and inflammatory dysfunction in coronary artery disease. J Exp Med. (2016) 213:337–54. doi: 10.1084/jem.20150900, PMID: 26926996 PMC4813677

[52] Umar S Palasiewicz K Volin MV Romay B Rahat R Tetali C . Metabolic regulation of RA macrophages is distinct from RA fibroblasts and blockade of glycolysis alleviates inflammatory phenotype in both cell types. Cell Mol Life Sci. (2021) 78:7693–707. doi: 10.1007/s00018-021-03978-5, PMID: 34705053 PMC8739866

[53] Suwa Y Nagafuchi Y Yamada S Fujio K . The role of dendritic cells and their immunometabolism in rheumatoid arthritis. Front Immunol. (2023) 14:1161148. doi: 10.3389/fimmu.2023.1161148, PMID: 37251399 PMC10213288

[54] Ashton MP Eugster A Dietz S Loebel D Lindner A Kuehn D . Association of Dendritic cell signatures with autoimmune inflammation revealed by Singleedtio profiling. Arthritis Rheumatol. (2019) 71:817–28. doi: 10.1002/art.40793, PMID: 30511817

[55] Henry Ó.C O’neill LA . Metabolic reprogramming in stromal and immune cells in rheumatoid arthritis and osteoarthritis: therapeutic possibilities. Eur J Immunol. (2025) 55:e202451381. doi: 10.1002/eji.202451381, PMID: 40170391 PMC11962241

[56] Bai Z Yang S Ren J Zhang C Chen X Huang H . Exploring the differential functions of circulating follicular helper T and peripheral helper T cells in rheumatoid arthritis based on metabolism patterns. Front Immunol. (2025) 16:1608675. doi: 10.3389/fimmu.2025.1608675, PMID: 40599793 PMC12209185

[57] Liu R Wu Q Su D Che N Chen H Geng L . A regulatory effect of IL-21 on T follicular helper-like cell and B cell in rheumatoid arthritis. Arthritis Res Ther. (2012) 14:R255. doi: 10.1186/ar3707, PMID: 23176102 PMC3674600

[58] Wen Z Jin K Shen Y Yang Z Li Y Wu B . N-myristoyltransferase deficiency impairs activation of kinase AMPK and promotes synovial tissue inflammation. Nat Immunol. (2019) 20:313–25. doi: 10.1038/s41590-018-0296-7, PMID: 30718913 PMC6396296

[59] Liang Y Zha M Liu Q Lai Z Li L Shao Y . Rheumatoid arthritis therapy based on B cells. Drug Design Dev Ther. (2025) 19:7837–52. doi: 10.2147/DDDT.S527687, PMID: 40951695 PMC12423268

[60] Carter LM Ehrenstein MR Vital EM . Evolution and trajectory of B-cell targeted therapies in rheumatic diseases. Lancet Rheumatol. (2025) 7:e355–67. doi: 10.1016/S2665-9913(24)00338-2, PMID: 40058377

[61] Takahashi S Saegusa J Sendo S Okano T Akashi K Irino Y . Glutaminase 1 plays a key role in the cell growth of fibroblast-like synoviocytes in rheumatoid arthritis. Arthritis Res Ther. (2017) 19:76. doi: 10.1186/s13075-017-1283-3, PMID: 28399896 PMC5387190

[62] Weyand CM Goronzy JJ . Immunometabolism in the development of rheumatoid arthritis. Immunol Rev. (2020) 294:177–87. doi: 10.1111/imr.12838, PMID: 31984519 PMC7047523

[63] Mangal JL Basu N Wu H-JJ Acharya AP . Immunometabolism: an emerging target for immunotherapies to treat rheumatoid arthritis. Immunometabolism. (2021) 3:e210032. doi: 10.20900/immunometab20210032

[64] Canavan M Walsh AM Bhargava V Wade SM Mcgarry T Marzaioli V . Enriched Cd141+ DCs in the joint are transcriptionally distinct, activated, and contribute to joint pathogenesis. JCI Insight. (2018) 3:e95228. doi: 10.1172/jci.insight.95228, PMID: 30518680 PMC6328029

[65] Weyand CM Goronzy JJ . Immunometabolism in early and late stages of rheumatoid arthritis. Nat Rev Rheumatol. (2017) 13:291–301. doi: 10.1038/nrrheum.2017.49, PMID: 28360422 PMC6820517

[66] Perl A . Review: metabolic control of immune system activation in rheumatic diseases. Arthritis Rheumatol. (2017) 69:2259–70. doi: 10.1002/art.40223, PMID: 28841779 PMC5711528

[67] Xue M Wang H Campos F Jackson CJ March L . Rheumatoid arthritis: biomarkers and the latest breakthroughs. Int J Mol Sci. (2025) 26:10594. doi: 10.3390/ijms262110594, PMID: 41226627 PMC12607731

[68] Puentes-Osorio Y Amariles P Calleja MÁ. Merino V Díaz-Coronado JC Taborda D . Potential clinical biomarkers in rheumatoid arthritis with an omic approach. Autoimmun Highlights. (2021) 12:9. doi: 10.1186/s13317-021-00152-6, PMID: 34059137 PMC8165788

[69] Maeda K Yoshida K Nishizawa T Otani K Yamashita Y Okabe H . Inflammation and bone metabolism in rheumatoid arthritis: molecular mechanisms of joint destruction and pharmacological treatments. Int J Mol Sci. (2022) 23:2871. doi: 10.3390/ijms23052871, PMID: 35270012 PMC8911191

[70] Giollo A Salvato M Frizzera F Khalid K Di Luozzo L Capita M . Clinical application of synovial biopsy in noninflammatory and persistent inflammatory refractory rheumatoid arthritis. Ann Rheumatic Diseases. (2025) 85:91–102. doi: 10.1016/j.ard.2025.07.023, PMID: 40846588

[71] Small A Wechalekar MD . Synovial biopsies in inflammatory arthritis: precision medicine in rheumatoid arthritis. Expert Rev Mol Diagnostics. (2020) 20:315–25. doi: 10.1080/14737159.2020.1707671, PMID: 31865803

[72] Scirè CA Epis O Codullo V Humby F Morbini P Manzo A . Immunohistological assessment of the synovial tissue in small joints in rheumatoid arthritis: validation of a minimally invasive ultrasound-guided synovial biopsy procedure. Arthritis Res Ther. (2007) 9:R101. doi: 10.1186/ar2302, PMID: 17903238 PMC2212566

[73] Humby F Durez P Buch MH Lewis MJ Rizvi H Rivellese F . Rituximab versus tocilizumab in anti-TNF inadequate responder patients with rheumatoid arthritis (R4RA): 16-week outcomes of a stratified, biopsy-driven, multicentre, open-label, phase 4 randomised controlled trial. Lancet. (2021) 397:305–17. doi: 10.1016/S0140-6736(20)32341-2, PMID: 33485455 PMC7829614

[74] Ketabchi S Russo E Benucci M Infantino M Manfredi M Cassarà EAM . Biopsy-driven synovial pathophenotyping in RA: A new approach to personalized treatment. J Personalized Med. (2025) 15:622. doi: 10.3390/jpm15120622, PMID: 41440985 PMC12733851

[75] Lewis MJ Çubuk C Surace AE Sciacca E Lau R Goldmann K . Deep molecular profiling of synovial biopsies in the STRAP trial identifies signatures predictive of treatment response to biologic therapies in rheumatoid arthritis. Nat Commun. (2025) 16:5374. doi: 10.1038/s41467-025-60987-9, PMID: 40603860 PMC12223067

[76] Hur B Gupta VK Huang H Wright KA Warrington KJ Taneja V . Plasma metabolomic profiling in patients with rheumatoid arthritis identifies biochemical features predictive of quantitative disease activity. Arthritis Res Ther. (2021) 23:164. doi: 10.1186/s13075-021-02537-4, PMID: 34103083 PMC8185925

[77] Singh A Behl T Sehgal A Singh S Sharma N Naved T . Mechanistic insights into the role of B cells in rheumatoid arthritis. Int Immunopharmacol. (2021) 99:108078. doi: 10.1016/j.intimp.2021.108078, PMID: 34426116

[78] Hur B Gupta VK Oh M Zeng H Crowson CS Warrington KJ . Integrative multi-omic profiling in blood reveals distinct immune and metabolic signatures between ACPA-negative and ACPA-positive rheumatoid arthritis. Front Immunol. (2025) 16:1667662. doi: 10.3389/fimmu.2025.1667662, PMID: 41235232 PMC12605004

[79] Zhou J Chen J Hu C Xie Z Li H Wei S . Exploration of the serum metabolite signature in patients with rheumatoid arthritis using gas chromatography–mass spectrometry. J Pharm Biomed Anal. (2016) 127:60–7. doi: 10.1016/j.jpba.2016.02.004, PMID: 26879423

[80] Jutley GS Sahota K Sahbudin I Filer A Arayssi T Young SP . Relationship between inflammation and metabolism in patients with newly presenting rheumatoid arthritis. Front Immunol. (2021) 12:676105. doi: 10.3389/fimmu.2021.676105, PMID: 34650548 PMC8507469

[81] Wang Q Ren J Lin X Zhang B Li J Weng Y . Inflammatory stimulus-responsive polymersomes reprogramming glucose metabolism mitigates rheumatoid arthritis. Biomaterials. (2025) 312:122760. doi: 10.1016/j.biomaterials.2024.122760, PMID: 39163825

[82] Sweeney SR Kavanaugh A Lodi A Wang B Boyle D Tiziani S . Metabolomic profiling predicts outcome of rituximab therapy in rheumatoid arthritis. RMD Open. (2016) 2:e000289. doi: 10.1136/rmdopen-2016-000289, PMID: 27651926 PMC5013418

[83] Gosselt HR Muller IB Jansen G Van Weeghel M Vaz FM Hazes JM . Identification of metabolic biomarkers in relation to methotrexate response in early rheumatoid arthritis. J personalized Med. (2020) 10:271. doi: 10.3390/jpm10040271, PMID: 33321888 PMC7768454

[84] Song X Lin Q . Genomics, transcriptomics and proteomics to elucidate the pathogenesis of rheumatoid arthritis. Rheumatol Int. (2017) 37:1257–65. doi: 10.1007/s00296-017-3732-3, PMID: 28493174

[85] Mcgarry T Hanlon MM Marzaioli V Cunningham CC Krishna V Murray K . Rheumatoid arthritis CD14+ monocytes display metabolic and inflammatory dysfunction, a phenotype that precedes clinical manifestation of disease. Clin Trans Immunol. (2021) 10:e1237. doi: 10.1002/cti2.1237, PMID: 33510894 PMC7815439

[86] Wang X Wang F Iyer AS Knight H Duggan LJ Yang Y . Integrative spatial proteomics and single-cell RNA sequencing unveil molecular complexity in rheumatoid arthritis for novel therapeutic targeting. Proteomes. (2025) 13:17. doi: 10.3390/proteomes13020017, PMID: 40559990 PMC12196869

[87] Centola M Cavet G Shen Y Ramanujan S Knowlton N Swan KA . Development of a multi-biomarker disease activity test for rheumatoid arthritis. PloS One. (2013) 8:e60635. doi: 10.1371/journal.pone.0060635, PMID: 23585841 PMC3621826

[88] Luan H Gu W Li H Wang Z Lu L Ke M . Serum metabolomic and lipidomic profiling identifies diagnostic biomarkers for seropositive and seronegative rheumatoid arthritis patients. J Trans Med. (2021) 19:1–10. doi: 10.1186/s12967-021-03169-7, PMID: 34876179 PMC8650414

[89] Baker JF Wipfler K Olave M Pedro S Katz P Michaud K . Obesity, adipokines, and chronic and persistent pain in rheumatoid arthritis. J Pain. (2023) 24:1813–9. doi: 10.1016/j.jpain.2023.05.008, PMID: 37207978

[90] Fortea-Gordo P Nuño L Villalba A Peiteado D Monjo I Sánchez-Mateos P . Two populations of circulating PD-1hiCD4 T cells with distinct B cell helping capacity are elevated in early rheumatoid arthritis. Rheumatology. (2019) 58:1662–73. doi: 10.1093/rheumatology/kez169, PMID: 31056653

[91] Jun-Jie J Meng-Ru G Hong M-J Jie S Zhouli S Dongyi H . Analysis of circulating immune cells in patients with rheumatoid arthritis and positive for hashimoto thyroiditis antibodies: Immune cells in RA & Hashimoto’s patients. Autoimmunity. (2025) 58:2445537. doi: 10.1080/08916934.2024.2445537, PMID: 41410036

[92] Masoumi M Hashemi N Moadab F Didehdar M Farahani R Khorramdelazad H . Immunometabolism dysfunction in the pathophysiology and treatment of rheumatoid arthritis. Curr Medicinal Chem. (2023) 30:3119–36. doi: 10.2174/0929867329666220907151213, PMID: 36082869

[93] Mangal JL . Designing metabolite-based therapies to rewire immunometabolism and treat autoimmune rheumatoid arthritis. Arizona State University (2022) Available online at: https://www.proquest.com/openview/01562218ad03863c41f23ef6431b8e91/1?pq-origsite=gscholar&cbl=18750&diss=y (Accesed July 11, 2025).

[94] Kim J-W Choe J-Y Park S-H . Metformin and its therapeutic applications in autoimmune inflammatory rheumatic disease. Korean J Internal Med. (2021) 37:13. doi: 10.3904/kjim.2021.363, PMID: 34879473 PMC8747910

[95] Rajaei E Haybar H Mowla K Zayeri ZD . Metformin one in a million efficient medicines for rheumatoid arthritis complications: Inflammation, osteoblastogenesis, cardiovascular disease, Malignancies. Curr Rheumatol Rev. (2019) 15:116–22. doi: 10.2174/1573397114666180717145745, PMID: 30019648

[96] Aiad AAE El-Haggar SM El-Barbary AM El-Afify DR . Metformin as adjuvant therapy in obese knee osteoarthritis patients. Inflammopharmacology. (2024) 32:2349–59. doi: 10.1007/s10787-024-01495-y, PMID: 38869746 PMC11300470

[97] Abed MS Aziz MZ Abdelhamid NM Soliman ES . Effects of metformin phonophoresis and exercise therapy on pain, range of motion, and physical function in chronic knee osteoarthritis: randomized clinical trial. J Orthopaedic Surg Res. (2024) 19:689. doi: 10.1186/s13018-024-05120-0, PMID: 39456024 PMC11515155

[98] Pan F Wang Y Lim YZ Urquhart DM Estee MM Wluka AE . Metformin for knee osteoarthritis in patients with overweight or obesity: a randomized clinical trial. JAMA. (2025) 333:1804–12. doi: 10.1001/jama.2025.3471, PMID: 40274279 PMC12022862

[99] Zhang R Lin X Lin R Chen Z Miao C Wang Y . Effectively alleviate rheumatoid arthritis via maintaining redox balance, inducing macrophage repolarization and restoring homeostasis of fibroblast-like synoviocytes by metformin-derived carbon dots. J Nanobiotechnology. (2025) 23:58. doi: 10.1186/s12951-025-03159-7, PMID: 39881361 PMC11776225

[100] Gharib M Elbaz W Darweesh E Sabri NA Shawki MA . Efficacy and safety of metformin use in rheumatoid arthritis: a randomized controlled study. Front Pharmacol. (2021) 12:726490. doi: 10.3389/fphar.2021.726490, PMID: 34630103 PMC8493211

[101] Abdallah MS Alarfaj SJ Saif DS El-Naggar ME Elsokary MA Elsawah HK . The AMPK modulator metformin as adjunct to methotrexate in patients with rheumatoid arthritis: A proof-of-concept, randomized, double-blind, placebo-controlled trial. Int Immunopharmacol. (2021) 95:107575. doi: 10.1016/j.intimp.2021.107575, PMID: 33773207

[102] Parab A Bhatt LK . T-cell metabolism in rheumatoid arthritis: focus on mitochondrial and lysosomal dysfunction. Immunopharmacol Immunotoxicology. (2024) 46:378–84. doi: 10.1080/08923973.2024.2330645, PMID: 38478010

[103] Zuo J Tang J Lu M Zhou Z Li Y Tian H . Glycolysis rate-limiting enzymes: novel potential regulators of rheumatoid arthritis pathogenesis. Front Immunol. (2021) 12:779787. doi: 10.3389/fimmu.2021.779787, PMID: 34899740 PMC8651870

[104] Kraus FV . Novel understanding of CD8+ T-cell regulation in Rheumatoid Arthritis–deciphering the close interplay between inflammation, epigenetics, and metabolism. Heidelberg University Hospital Publisher (2025) Available online at: https://archiv.ub.uni-heidelberg.de/volltextserver/34466/ (Accesed July 19, 2025).

[105] Uehara I Kajita M Tanimura A Hida S Onda M Naito Z . 2022.A,A,.n resea induces deglycosylation of proinflammatory cytokine receptors and strongly reduces immunological responses in mouse models of inflammation. Pharmacol Res Perspect. (2022) 10:e00940. doi: 10.1002/prp2.940, PMID: 35212163 PMC8873284

[106] Wang H Zhang N Fang K Chang X . 2-Deoxy-D-glucose alleviates collagen-induced arthritis of rats and is accompanied by metabolic regulation of the spleen and liver. Front Immunol. (2021) 12:713799. doi: 10.3389/fimmu.2021.713799, PMID: 34539643 PMC8440946

[107] Friedman B Cronstein B . Methotrexate mechanism in treatment of rheumatoid arthritis. Joint Bone Spine. (2019) 86:86. doi: 10.1016/j.jbspin.2018.07.004, PMID: 30081197 PMC6360124

[108] Katturajan R Vijayalakshmi S Rasool M Prince SE . Molecular toxicity of methotrexate in rheumatoid arthritis treatment: A novel perspective and therapeutic implications. Toxicology. (2021) 461:152909. doi: 10.1016/j.tox.2021.152909, PMID: 34453959

[109] Fujii T Atsumi T Okamoto N Takahashi N Tamura N Nakajima A . AB0249 safety of baricitinib in Japanese patients with rheumatoid arthritis (RA): The 2020 interim report from all-case post marketing surveillance in clinical practice. Ann Rheumatic Dis. (2021) 80:1150. doi: 10.1136/annrheumdis-2021-eular.433, PMID: 41686241

[110] Solomon DH Glynn RJ Karlson EW Lu F Corrigan C Colls J . Adverse effects of low-dose methotrexate: a randomized trial. Ann Internal Med. (2020) 172:369–80. doi: 10.7326/M19-3369, PMID: 32066146 PMC7229518

[111] Lee EB Fleischmann R Hall S Wilkinson B Bradley JD Gruben D . Tofacitinib versus methotrexate in rheumatoid arthritis. New Engl J Med. (2014) 370:2377–86. doi: 10.1056/NEJMoa1310476, PMID: 24941177

[112] Khokhar M Dey S Tomo S Jaremko M Emwas A-H Pandey RK . Unveiling novel drug targets and emerging therapies for rheumatoid arthritis: A comprehensive review. ACS Pharmacol Trans Sci. (2024) 7:1664–93. doi: 10.1021/acsptsci.4c00067, PMID: 38898941 PMC11184612

[113] Behl T Chadha S Sehgal A Singh S Sharma N Kaur R . Exploring the role of cathepsin in rheumatoid arthritis. Saudi J Biol Sci. (2022) 29:402–10. doi: 10.1016/j.sjbs.2021.09.014, PMID: 35002435 PMC8716961

[114] Zezina E Sercan Revi O Herrmann M Biesemann N . Glucose transporter 1 in rheumatoid arthritis and autoimmunity. Wiley Interdiscip Reviews: Syst Biol Med. (2020) 12:e1483. doi: 10.1002/wsbm.1483, PMID: 32084302

[115] Michalek RD Gerriets VA Jacobs SR Macintyre AN Maciver NJ Mason EF . Cutting edge: distinct glycolytic and lipid oxidative metabolic programs are essential for effector and regulatory CD4+ T cell subsets. J Immunol. (2011) 186:3299–303. doi: 10.4049/jimmunol.1003613, PMID: 21317389 PMC3198034

[116] Okano T Saegusa J Takahashi S Ueda Y Morinobu A . Immunometabolism in rheumatoid arthritis. Immunol Med. (2018) 41:89–97. doi: 10.1080/25785826.2018.1531186, PMID: 30938274

[117] Ahmed S Mahony CB Torres A Murillo-Saich J Kemble S Cedeno M . Dual inhibition of glycolysis and glutaminolysis for synergistic therapy of rheumatoid arthritis. Arthritis Res Ther. (2023) 25:176. doi: 10.1186/s13075-023-03161-0, PMID: 37730663 PMC10510293

[118] Zhao Y Yan X Li X Zheng Y Li S Chang X . PGK1, a glucose metabolism enzyme, may play an important role in rheumatoid arthritis. Inflammation Res. (2016) 65:815–25. doi: 10.1007/s00011-016-0965-7, PMID: 27342824

[119] Panneton V Bagherzadeh Yazdchi S Witalis M Chang J Suh WK . and maintenance of collagen-induced arthritis. J Immunol. (2018) 200:3067–76. doi: 10.4049/jimmunol.1701305, PMID: 29581356

[120] Bian L Josefsson E Jonsson IM Verdrengh M Ohlsson C Bokarewa M . Dichloroacetate alleviates development of collagen II-induced arthritis in female DBA/1 mice. Arthritis Res Ther. (2009) 11:R132. doi: 10.1186/ar2799, PMID: 19723321 PMC2787291

[121] Zhang Y Jin H Jia W Liu Y Wang Y Xue S . Ermiao San attenuating rheumatoid arthritis via PI3K/AKT/mTOR signaling activate HIF-1α induced glycolysis. J Ethnopharmacology. (2025) 345:119615. doi: 10.1016/j.jep.2025.119615, PMID: 40081512

[122] He Y-F Mai C-T Pan H-D Liu L Zhou H Xie Y . Targeting immunometabolism by active ingredients derived from traditional Chinese medicines for treatment of rheumatoid arthritis. Chin Herbal Medicines. (2021) 13:451–60. doi: 10.1016/j.chmed.2021.09.005, PMID: 36119361 PMC9476673

[123] Cheng J-W Yu Y Zong S-Y Cai W-W Wang Y Song Y-N . Berberine ameliorates collagen-induced arthritis in mice by restoring macrophage polarization via AMPK/mTORC1 pathway switching glycolytic reprogramming. Int Immunopharmacol. (2023) 124:111024. doi: 10.1016/j.intimp.2023.111024, PMID: 37827054

[124] Cai W-W Gao Y Cheng J-W Yu Y Zong S-Y Li Y-H . Berberine modulates the immunometabolism and differentiation of CD4+ T cells alleviating experimental arthritis by suppression of M1-exo-miR155. Phytomedicine. (2024) 124:155255. doi: 10.1016/j.phymed.2023.155255, PMID: 38181528

[125] Sheng S Wang X Liu X Hu X Shao Y Wang G . The role of resveratrol on rheumatoid arthritis: From bench to bedside. Front Pharmacol. (2022) 13:829677. doi: 10.3389/fphar.2022.829677, PMID: 36105210 PMC9465647

[126] Wang X Khalil RA . Matrix metalloproteinases, vascular remodeling, and vascular disease. Adv Pharmacol. (2018) 81:241–330. doi: 10.1016/bs.apha.2017.08.002, PMID: 29310800 PMC5765875

[127] Goetz LH Schork NJ . Personalized medicine: motivation, challenges, and progress. Fertility sterility. (2018) 109:952–63. doi: 10.1016/j.fertnstert.2018.05.006, PMID: 29935653 PMC6366451

[128] Tavakolpour S . Towards personalized medicine for patients with autoimmune diseases: opportunities and challenges. Immunol Lett. (2017) 190:130–8. doi: 10.1016/j.imlet.2017.08.002, PMID: 28797806

[129] Bhamidipati K Wei K . Precision medicine in rheumatoid arthritis. Best Pract Res Clin Rheumatol. (2022) 36:101742. doi: 10.1016/j.berh.2022.101742, PMID: 35248489 PMC8977251

[130] Madrid-Paredes A Martín J Márquez A . -Omic approaches and treatment response in rheumatoid arthritis. Pharmaceutics. (2022) 14:1648. doi: 10.3390/pharmaceutics14081648, PMID: 36015273 PMC9412998

[131] Müller R Kull M Põlluste K Aart A Eglit T Lember M . The metabolic profile in early rheumatoid arthritis: a high prevalence of metabolic obesity. Rheumatol Int. (2017) 37:21–7. doi: 10.1007/s00296-016-3464-9, PMID: 27084374

[132] Medcalf MR Bhadbhade P Mikuls TR O’dell JR Gundry RL Funk RS . Plasma metabolome normalization in rheumatoid arthritis following initiation of methotrexate and the identification of metabolic biomarkers of efficacy. Metabolites. (2021) 11:824. doi: 10.3390/metabo11120824, PMID: 34940582 PMC8706490

[133] Ding Z Chen W Wu H Li W Mao X Su W . Integrative network fusion-based multi-omics study for biomarker identification and patient classification of rheumatoid arthritis. Chin Med. (2023) 18:48. doi: 10.1186/s13020-023-00750-8, PMID: 37143094 PMC10158004

[134] Ghanouni M . The role of immunometabolism in disease. In: Development engineering conferences center articles database Development Engineering Conferences Center (2024) Available online at: https://pubs.bcnf.ir/index.php/Articles/article/view/52 (Accesed July 19, 2025).

[135] Lu Y Wang Y Ruan T Wang Y Ju L Zhou M . Immunometabolism of Tregs: mechanisms, adaptability, and therapeutic implications in diseases. Front Immunol. (2025) 16:1536020. doi: 10.3389/fimmu.2025.1536020, PMID: 39917294 PMC11798928

[136] Schiotis R Buzoianu A Mureşanu D Suciu S . New pharmacological strategies in rheumatic diseases. J Med Life. (2016) 9:227 Available online at: https://pmc.ncbi.nlm.nih.gov/articles/PMC5154305/ (Accesed July 20, 2025). 27974925 PMC5154305

[137] Keskitalo S Seppänen M Del Sol A Varjosalo M . From rare to more common: The emerging role of omics in improving understanding and treatment of severe inflammatory and hyperinflammatory conditions. J Allergy Clin Immunol. (2025) 155:1435–50. doi: 10.1016/j.jaci.2025.02.011, PMID: 39978687

[138] Xu R He X Xu J Yu G Wu Y . Immunometabolism: signaling pathways, homeostasis, and therapeutic targets. MedComm. (2024) 5:e789. doi: 10.1002/mco2.789, PMID: 39492834 PMC11531657

[139] Le Naour J Galluzzi L Zitvogel L Kroemer G Vacchelli E . Trial watch: IDO inhibitors in cancer therapy. Oncoimmunology. (2020) 9:1777625. doi: 10.1080/2162402X.2020.1777625, PMID: 32934882 PMC7466863

[140] Hegarty C Neto N Cahill P Floudas A . Computational approaches in rheumatic diseases–Deciphering complex spatio-temporal cell interactions. Comput Struct Biotechnol J. (2023) 21:4009–20. doi: 10.1016/j.csbj.2023.08.005, PMID: 37649712 PMC10462794

[141] Fujii W Kawahito Y Nagahara H Kukida Y Seno T Yamamoto A . Monocarboxylate transporter 4, associated with the acidification of synovial fluid, is a novel therapeutic target for inflammatory arthritis. Arthritis Rheumatol. (2015) 67:2888–96. doi: 10.1002/art.39270, PMID: 26213210

[142] Dong Q Wu J Zhang H Chen X Xu X Chen J . Deciphering immunometabolic landscape in rheumatoid arthritis: integrative multiomics, explainable machine learning and experimental validation. J Inflammation Res. (2025) 18:637–52. doi: 10.2147/JIR.S503118, PMID: 39835297 PMC11745140

[143] Lewis DM Park KM Tang V Xu Y Pak K Eisinger-Mathason TK . Intratumoral oxygen gradients mediate sarcoma cell invasion. Proc Natl Acad Sci. (2016) 113:9292–7. doi: 10.1073/pnas.1605317113, PMID: 27486245 PMC4995943

[144] Weyand CM Goronzy JJ . Immunometabolism in the development of rheumatoid arthritis. Immunol Rev. (2020) 294:177–87. doi: 10.1111/imr.12838, PMID: 31984519 PMC7047523

[145] Suwa Y Nagafuchi Y Yamada S Fujio K . The role of dendritic cells and their immunometabolism in rheumatoid arthritis. Front Immunol. (2023) 14:1161148. doi: 10.3389/fimmu.2023.1161148, PMID: 37251399 PMC10213288

[146] Weyand CM Goronzy JJ . Immunometabolism in early and late stages of rheumatoid arthritis. Nat Rev Rheumatol. (2017) 13:291–301. doi: 10.1038/nrrheum.2017.49, PMID: 28360422 PMC6820517

[147] Luo T-T Wu Y-J Yin Q Chen W-G Zuo J . The involvement of glucose and lipid metabolism alteration in rheumatoid arthritis and its clinical implication. J Inflammation Res. (2023) 16:1837–52. doi: 10.2147/JIR.S398291, PMID: 37131409 PMC10149064

[148] Shakeel L Shaukat A Khaliq N Kashif A Mujeeb A Adnan Z . Rheumatoid arthritis: a comprehensive overview of genetic markers, emerging therapies, and personalized medicine. Ann Med Surg. (2025) 87:696–710. doi: 10.1097/MS9.0000000000002890, PMID: 40110258 PMC11918739

[149] Song Y Milichko VA Ding Z Li W Kang B Dou Y . Double crosseVA,.gy hydrogel for intragel,.gy in injection as modality for macrophages metabolic reprogramming and therapy of rheumatoid arthritis. Advanced Funct Materials. (2025) 2502880. doi: 10.1002/adfm.202502880, PMID: 41773552

[150] Martinsson K Dürholz K Schett G Zaiss MM Kastbom A . Higher serum levels of short-chain fatty acids are associated with non-progression to arthritis in individuals at increased risk of RA. Ann Rheumatic Dis. (2022) 81:445–7. doi: 10.1136/annrheumdis-2021-221386, PMID: 34819270 PMC8862054

[151] Wei Q Qian Y Yu J Wong CC . Metabolic rewiring in the promotion of cancer metastasis: mechanisms and therapeutic implications. Oncogene. (2020) 39:6139–56. doi: 10.1038/s41388-020-01432-7, PMID: 32839493 PMC7515827

[152] Gao Y Zhang Y Liu X . Rheumatoid arthritis: pathogenesis and therapeutic advances. MedComm. (2024) 5:e509. doi: 10.1002/mco2.509, PMID: 38469546 PMC10925489

[153] Komatsu N Takayanagi H . Mechanisms of joint destruction in rheumatoid arthritis—immune cell–fibroblast–bone interactions. Nat Rev Rheumatol. (2022) 18:415–29. doi: 10.1038/s41584-022-00793-5, PMID: 35705856

[154] O’neil LJ Alpízar-Rodríguez D Deane KD . Rheumatoid arthritis: the continuum of disease and strategies for prediction, early intervention, and prevention. J Rheumatol. (2024) 51:337–49. doi: 10.3899/jrheum.2023-0334, PMID: 38224993 PMC10984790

